# Durable Modification of Wood by Benzoylation—Proof of Covalent Bonding by Solution State NMR and DOSY NMR Quick-Test †

**DOI:** 10.3390/polym13132164

**Published:** 2021-06-30

**Authors:** Jan C. Namyslo, Martin H. H. Drafz, Dieter E. Kaufmann

**Affiliations:** Institute of Organic Chemistry, Clausthal University of Technology, Leibnizstr. 6, 38678 Clausthal-Zellerfeld, Germany; jan.namyslo@tu-clausthal.de (J.C.N.); M.Drafz@t-online.de (M.H.H.D.)

**Keywords:** covalent wood modification, esterification, wood dissolution, solution state NMR, non-uniform sampling (NUS), HMBC, DOSY

## Abstract

A convenient, broadly applicable and durable wood protection was recently published by Kaufmann and Namyslo. This procedure efficiently allows for esterification of wood hydroxyl groups with (1*H*-benzotriazolyl)-activated functionalized benzoic acids. The result of such wood-modifying reactions is usually monitored by an increase in mass of the wood material (weight percent gain value, WPG) and by infrared spectroscopy (IR). However, diagnostic IR bands suffer from overlap with naturally occurring ester groups, mainly in the hemicellulose part of unmodified wood. In contrast to known NMR spectroscopy approaches that use the non-commonly available solid state techniques, herein we present solution state NMR proof of the covalent attachment of our organic precursors to wood. The finding is based on a time-efficient, non-uniformly sampled (NUS) solution state ^1^H,^13^C-HMBC experiment that only needs a tenth of the regular recording time. The appropriate NMR sample of thoroughly dissolved modified wood was prepared by a mild and non-destructive method. The 2D-HMBC shows a specific cross-signal caused by spin–spin coupling over three bonds from the ester carbonyl carbon atom to the α-protons of the esterified wood hydroxyl groups. This specific coupling pathway requires a covalent bonding as a *conditio sine qua non*. An even more rapid test to monitor the covalent bonding was achieved with an up-to-date diffusion-ordered spectroscopy sequence (Oneshot—DOSY) based on ^1^H or ^19^F as the sensitive nucleus. The control experiment in a series of DOSY spectra gave a by far higher D value of (1.22 ± 0.06)∙10^−10^ m^2^∙s^−1^, which is in accordance with fast diffusion of the “free” and thus rapidly moving small precursor molecule provided as its methyl ester. In the case of a covalent attachment to wood, a significantly smaller D value of (0.12 ± 0.01)∙10^−10^ m^2^∙s^−1^ was obtained.

## 1. Introduction

Classic wood treatment methods that involve penetration, but later on also allow release of the applied inorganic or appropriately functionalized organic compounds have become more and more obsolete as non-sustainable and environmentally benign procedures. Hence, recent developments in the field of chemical wood modification have focused on the formation of strong chemical bonds between accessible wood hydroxyl groups and appropriate functionalizing compounds [1,2]. This way, a long-time durability of the protected natural material is ensured. Such material improvements in most cases aim at a decrease of deterioration by water or UV exposure, fungi, voracious termites, or other insects. Additional improvements such as flame retardancy are in demand and have been put into practice by this concept of covalent attachment of functional organic precursors. Thus far, after initial chemical and analytical investigations in the field of common wood acetylation, we have focused on multipurpose benzoylations applying boron-, carbon-, silicon-, nitrogen-, phosphorus-, and halogen-functionalized activated benzamides as starting materials for the modification (de facto esterification) of wood hydroxyl groups [3,4,5,6,7,8,9].

Due to the complex structure of wood and its main constituents lignin, cellulose, and hemicellulose, the subsequent structural analysis of the chemically modified material remains difficult, although for about half a century, the formation of an ester moiety has been generally known and accepted on account of the chemical reaction between wood hydroxyl groups and an appropriate activated organic acid or a corresponding acid anhydride, respectively.

The actual ester formation, thereby the extent of covalent bonding to wood, is usually analyzed by gravimetric determination of the so-called weight percent gain (WPG), a value that is well established in wood chemistry [1,2,3,10]. Reflected by a positive WPG value, even after subsequent washing and extraction steps, the resulting increase in mass clearly adverts to a durable type of wood modification as it is known for covalent bonding. 

Even though further analysis of the chemically modified wood in comparison to the natural material includes IR spectroscopy [3,11,12,13,14,15], this analytical technique cannot be used to unquestionably prove a covalent bond between the modifying organic compound and wood due to the signal overlap that masks the most diagnostic carbonyl ester band at about 1740 to 1720 wave numbers. Such a C=O stretching vibration does not exclusively appear upon formation of the newly formed ester but is detected as a simply growing signal, among many that are originally present in wood (e.g., from partially acetylated hemicellulose). 

In order to solve the question of whether this covalent bonding to wood hydroxyl groups was successful, the spectroscopic method of choice is nuclear magnetic resonance, NMR. In the present case, even though the widespread solution state NMR de facto is a fast and unbeatable analytical tool for small organic molecules dissolved in an appropriate deuterated NMR solvent, of course the natural polymer wood suffers from its at best marginal solubility on the way to an appropriate solution state NMR sample. Nevertheless, the solution state NMR approach has been pursued further, as the solid state counterpart is available only to a quite fractional part of all chemistry labs. To analyze a sample of the entire covalently modified wood by means of solution state NMR, a suitable preparation technique has to be applied. With such a sample, an HMBC experiment—a common technique for small molecules—was performed within some ten minutes. However, in the present case, this 2D NMR experiment was only feasible upon application of non-uniform data sampling (NUS) [16,17,18] with the recording of a 10 percent fraction of data that allowed a measurement time of about four days. This experiment resulted in the confirmation of a covalent-type bonding of such benzoylation reagents to wood, but the still high expenditure of time prompted us to additionally aim toward a serviceable day-to-day method, again utilizing commonly available solution state NMR probe heads. 

Keeping this in mind, we found that solution state diffusion-ordered spectroscopy (DOSY) [19,20,21,22,23,24,25,26,27,28] is a valuable technique well-known from organic macromolecules such as technical polymers. It should be an appropriate tool to distinguish between fast diffusing small precursor molecules from slow moving wood, which has been modified with the functionalized organic precursor. In this field of wood chemistry and structural analysis, up until now, there have only been a few papers that utilize the DOSY technique, and among these, solely fractionated material (lignin) has been investigated [29,30,31] or esterified cellulose [32].

In the course of our investigations, we aimed at the solution state NMR spectroscopic analysis of entire wood in its natural form, and in comparison, chemically modified. In contrast to papers that involve single constituents of wood (e.g., (hemi)cellulose or lignin or even partly degraded components), it appears that such papers are expectedly rare due to the limited solubility of the whole material. Early research, for example, by Ohkoshi and co-workers, solely makes use of DMSO soluble portions (not the entire material) or is based on chemically preprocessed (delignified) wood samples, which were then dissolved in solvent mixtures with *N*-methylmorpholine *N*-oxide monohydrate [33,34,35,36]. In order to make any kind of wood sample accessible to solution state NMR, often times harsh conditions have been used for the upstream dissolution process [37]. Hence, at least partial degradation appears or a type of preprocessing of the wood leads to single constituents (i.e., lignin and (processed) parts thereof or cell walls) [38,39,40,41,42,43]. In several cases, solution state NMR has been performed with milled wood lignin (MWL) [44,45,46,47,48,49,50,51,52], residual and Kraft lignin [46,53,54], or (hemi)cellulosic fractions of wood, which were in this way spectroscopically investigated [37,55]. 

A totally different approach known from the literature is the dissolution of wood and its constituents or other type of ‘biomass’ by means of ionic liquids (ILs) [37,56,57,58,59]. Either the IL is used for the dissolution of wood in order to subsequently acylate and then recover the thus modified material [57,58,59], or the IL itself acts as a solvent that is appropriate for the NMR measurement [60,61]. The latter case suffers from restricted practicability due to a number of strong ^1^H-NMR signals of the proton bearing IL itself. Therefore, only chemical shift areas that are free from the IL signals are suitable [37]. As an alternative, especially in proton NMR or proton based inverse 2D NMR spectroscopy, more or less laborious syntheses of deuterated counterparts of such ILs have been published [62,63]. Another approach is the use of an IL as co-solvent with, for example, common DMSO-*d_6_* [43]. The use of supporting chemicals such as pyridinium chloride [40] can be considered as a preliminary stage of IL application. In addition, the parent compound pyridine has been found as solvent or co-solvent in the literature (e.g., as a 1.6:1 mixture with CDCl_3_ [64] or with DMSO-*d_6_*) [41]. Another precursor that is much closer to the concept of IL application is *N*-methyl imidazole (NMI) [38]. Despite this potpourri of known solvents and cosolvents, a consistently appropriate solvent for solution state NMR in the field of wood analysis is hard to find. Solely due to the thus restricted solubility, most NMR studies involve the solid state technique. Since the beginning of the 1980s, more than 500 papers (until May 2021) have been published and efforts in this field have been reviewed from time to time (e.g., by Nimz et al. [65], Gagnaire [66], Gil and Pascoal Neto [67], Frazier and co-workers [68], Atalla and VanderHart [69], Maunu [70], and Wikberg and Maunu [71]). More recently, comprehensive papers have appeared by Bardet et al. [72], Ralph and Landucci [73], and Terrett et al. [74]. In some cases, solid state and at the same time, solution (also referred to as liquid) state NMR, have been applied in this research field [75,76,77,78,79,80]. 

Regarding the efficiency of solid state NMR, note that even in 2021, most of these spectrometers are far away from fast (around 100 kHz) or ultrafast magic angle spinning (MAS) frequencies such as 150 kHz MAS, which was very recently published in the field of other biomacromolecules, in particular, protein analysis by Schledorn et al. [81]. However, until actually fast or even such ultrafast spinning frequencies that significantly reduce the former discrepancy in spectral resolution compared to solution state NMR spectroscopy (with from fast molecular tumbling naturally born single-type isotropic and narrow shaped NMR shifts) are widely accessible, we assume that the solution state counterpart will be the method of choice whenever a dissolved sample can be prepared. Thereby, ongoing spectrometer development in the GHz class (i.e., commercially available 1.2 GHz in 2020 [82]) benefits both techniques. 

All in all, as our chemistry lab, and also by far most others are equipped with solution state NMR spectrometers, our own attempts exclusively focused on this equipment. Hence, herein we present a very mild, thus non-destructive preparation procedure in order to provide solution state NMR samples of wood that were chemically improved via benzoylation with appropriate functionalizing precursor molecules. The solvent system suitable in our case for the chemically modified wood was LiCl-doped DMSO-*d_6_* according to Wang et al. [83]. The sample preparation was accomplished within two hours and did not need sonication and/or centrifugation steps that would probably be necessary in case of a lower extent of benzoylation or in the case of chemically unmodified material [42]. In DMSO-*d_6_*/LiCl, the concentration of the dissolved benzoylated Scots pine (*Pinus sylvestris* L.) sapwood was still at the lower end of spectroscopic feasibility, but was experimentally compensated, recording otherwise time-consuming 2D spectra with the above-mentioned NUS technique (acquisition of only 10% of regular data). In addition to easily and fast accessible one dimensional proton spectra from such a sample, even a well resolved ^1^H,^13^C-HMBC spectrum was obtained by Drafz [84]. In this 2D solution state NMR spectrum, significant cross-signals were detected that unambiguously prove the existence of a covalent bond between wood and the functionalized reagent that was attached. In order to provide a quasi “attachment quick test”, we additionally performed a highly efficient variant of diffusion-ordered spectroscopy (Oneshot-DOSY) [25]. 

## 2. Materials and Methods

### 2.1. General Procedures

The organic compounds mentioned in this paper were structurally characterized by solution state NMR spectroscopy, mass spectrometry, and infrared spectroscopy. 

**Melting point apparatus:** Büchi 520 (Büchi, Flavil, Switzerland). The melting points were uncorrected. 

**NMR instrument:** ‘Bruker Avance III 600′ (Bruker Biospin, Rheinstetten, Germany) with 600 MHz proton frequency equipped with a room temperature BBO-probe head with z-Gradient. ^1^H-NMR spectra in CDCl_3_ were referenced to internal tetramethylsilane (TMS 0.0 ppm). ^13^C-NMR spectra refer to the solvent signal center at 77.0 ppm. In the case of DMSO-*d_6_*, the solvent peak was set to 2.50 ppm (^1^H) and 39.5 ppm (^13^C), respectively. ^15^N-NMR spectra refer to external CH_3_NO_2_ (0.0 ppm) and were measured at their appropriate resonance frequency on the aforementioned spectrometer. ^15^N chemical shifts were obtained from one-dimensional spectra in inverse-gated decoupling mode or from inverse-detected, two-dimensional ^1^H,^15^N-HMBC spectra. DOSY spectra (^1^H and ^19^F) were obtained on the same spectrometer, but applying a TXO (^1^H,^19^F,^13^C) probe head with z-Gradient. Calibration of the gradient strength G was conducted by means of a deuterium oxide sample containing H_2_O-traces [85]. ^19^F spectra were referenced to trichlorofluoromethane at 0.0 ppm. DOSY measurements were conducted in high quality 3 mm NMR tubes (Wilmad 335 in an adequate spinner provided with a mass-ring) under calibrated (‘methanol thermometer’) and carefully stabilized temperature conditions (i.e., 298 ± 0.1 K). The probe head nitrogen gas flow was adjusted to 800 l/h. Optimized pulse repetition delays (d1) were obtained from T1 inversion–recovery measurements. Sample spinning was used in order to avoid convection [86]. Prior to each DOSY experiment, repeated gradient shimming was performed. All NMR coupling constants are given in Hz. Multiplicities are described by using the following abbreviations: s = singlet, d = doublet, t = triplet, q = quartet, and m = multiplet. 

**IR instrument**: ‘Vector 22 FTIR’ (Bruker, Bremen, Germany) equipped with a ‘Specac Golden-Gate’ Diamond-ATR/KRS5 unit applying the samples as solids. 

**Mass spectra** were recorded on a ‘Varian 320 MS’ (Varian, Darmstadt, Germany) equipped with a triple quadrupole with direct inlet and electron impact ionization (EI, 20 eV). HRMS(ESI) was measured at the Institute of Organic Chemistry, Leibniz-University, Hannover, Germany, with a Waters Q-Tof Premier coupled with an Acquity UPLC equipment. 

### 2.2. Chemicals, Solvents, and Wood Materials

Dichloromethane (DCM) was dried using an “MP5 Solvent Purification System” (Innovative Technology, Amesbury, MA, USA). All other solvents were dried according to standard methods and were freshly distilled prior to use. Anhydrous (anhyd.) *N*,*N*-dimethylformamide (DMF) and all other chemicals were used as purchased from Sigma-Aldrich Chemie GmbH, Taufkirchen, Germany. Wood samples were obtained from the Section of Wood Biology and Wood Products, Georg-August-University Göttingen, Göttingen, Germany.

### 2.3. Synthesis of 1H-benzotriazol-1-yl(phenyl)methanone-α-^13^C


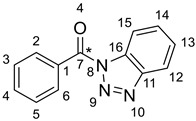


A solution of 1.50 g (12.6 mmol) 1*H*-benzotriazole and 0.3 mL (4.5 mmol) thionyl chloride in 10 mL anhyd. DCM was added dropwise to a suspension of 0.50 g (4.10 mmol) *α*-^13^C-benzoic acid in 20 mL anhyd. DCM within 30 min. Subsequently, the reaction mixture was stirred for 16 h at room temperature (r.t.). After hydrolysis with water (20 mL), the aqueous (aq) phase was extracted with DCM (1 × 30 mL). The combined organic layers were washed with water (5 × 100 mL) and then dried over anhyd. sodium sulfate. Evaporation of the solvent gave 0.779 g (85%) of pure product as a white solid. M.p.: 112 °C.

IR (ATR): $\bar{\nu }$ = 3103, 3068, 1665, 1599, 1580, 1487, 1449, 1375, 1354, 1319, 1305, 1286, 1237, 1225, 1181, 1154, 1132, 1107, 1093, 1048, 1035, 1004, 972,876, 787, 771, 751, 693, 678, 656, 633, 617, 583, 563, 529, 456, 433 cm^−1^. ^1^H NMR (600 MHz, CDCl_3_): *δ* = 8.35–8.31 (m, 1H, H-15), 8.21–8.17 (m, 2H, H-2, H-6), 8.13–8.10 (m, 1H, H-12), 7.67–7.62 (m, 2H, H-4, H-14), 7.56–7.51 (m, 2H, H-3, H-5), 7.51–7.47 (m, 1H, H-13) ppm. ^13^C NMR (150 MHz, CDCl_3_): *δ* = 166.5 (C_quat_, C-7), 145.6 (C_quat_, d, *J* = 3.4 Hz, C-11), 133.5 (+, C-14), 132.1 (C_quat_, C-16), 131.6 (+, d, *J* = 2.2 Hz, 2C, C-2, C-6), 131.3 (C_quat_, d, *J* = 69.3 Hz, C-1), 130.2 (+, C-4), 128.2 (+, d, *J* = 4.4 Hz, C-3, C-5), 126.1 (+, C-13), 120.0 (+, C-12), 114.6 (+, C-15) ppm. ^15^N-NMR (61 MHz, CDCl_3_): *δ* = 5.5 (d, *J* = 4.1 Hz, N-9), −18.9 (N-10), −123.2 (d, *J* = 11.4 Hz, N-8) ppm. EI-MS (20 eV); *m/z* (%): 224.2 [M^+^] (10), 196.1 [M-N_2_]^+^ (100), 106.0 [M-Bt]^+^ (80). HRMS (ESI): calcd.: 247.0678 (+Na), found: 247.0677 (Δ = 0.4 ppm).

### 2.4. Synthesis of 1H-benzotriazol-1-yl[3,5-bis(trifluoromethyl)phenyl]methanone


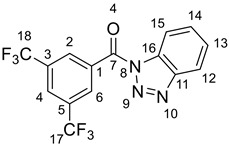


To a slurry of 5.00 g (19.4 mmol) 3,5-bis(trifluoromethyl)benzoic acid in 20 mL anhyd. DCM a solution of 7.15 g (60.1 mmol) 1*H*-benzotriazole and 1.6 mL (21.3 mmol) thionyl chloride in 20 mL of the same solvent was added dropwise within 30 min. The resulting mixture was stirred for 16 h at r.t. After the addition of water (20 mL), the aq phase was extracted with DCM (1 × 20 mL). Subsequently, the organic layer was washed with water (4 × 50 mL) and then dried over anhyd. magnesium sulfate. Evaporation of the solvent gave an almost pure product that was further purified via column chromatography (silica gel, DCM as the eluent). White solid, m.p.: 84 °C, 6.82 g, 98% yield. 

IR (ATR): $\bar{\nu }$ = 3125, 3047, 1698, 1615, 1599, 1486, 1454, 1386, 1347, 1326, 1274, 1210, 1180, 1128, 1099, 1043, 1009, 965, 921, 846, 788, 772, 757, 707, 694, 681, 640, 561, 432 cm^−1^. ^1^H NMR (600 MHz, CDCl_3_): *δ* = 8.73–8.70 (m, 2 H, H-2, H-6), 8.39 (ddd, *J* = 8.3, 1.0, 1.0 Hz, 1 H, H-15), 8.21–8.18 (m, 2 H, H-4, H-12), 7.76 (ddd, *J* = 8.3, 7.2, 1.0 Hz, 1 H, H-14), 7.61 (ddd, *J* = 8.3, 7.2, 1.0 Hz, 1 H, H-13) ppm. ^13^C NMR (150.97 MHz, CDCl_3_): *δ* = 163.9 (C_quat_, C-7), 145.9 (C_quat_, C-11), 133.6 (C_quat_, C-16), 132.2 (C_quat_, q, *J* = 34.1 Hz, 2 C, C-3, C-5), 132.0 (C_quat_, C-1), 131.7 (+, q, *J* = 3.3 Hz, 2 C, C-2, C-6), 131.1 (+, C-14), 127.0 (+, C-13), 126.8 (m, C-4), 122.8 (C_quat_, q, *J* = 272.2 Hz, 2 C, C-17, C-18), 120.6 (+, C-12), 114.7 (+, C-15) ppm. ^15^N-NMR (60.85 MHz, CDCl_3_): *δ* = 3.2 (N-9), −14.3 (N-10), −124.4 (N-8) ppm. ^19^F-NMR (564.83 MHz, CDCl_3_): *δ* = −62.9 ppm. EI-MS (20 eV); *m/z* (%): 359.1 [M^+^] (20), 331.1 [M-N_2_]^+^ (100), 241.1 [M-Bt]^+^ (95) [87].

### 2.5. Chemical Wood Modification Procedure

In general, a standard amount of 7.0 mmol of the appropriate wood modifying reagent is applied per gram of Scots pine (*Pinus sylvestris* L.) sapwood veneer chips of approx. dimensions 25 × 15 × 0.6 mm^3^ (ca. 0.100 g). In case of the 1*H*-benzotriazol-1-yl(phenyl)methanone-α-^13^C wood modification, an amount of 10.5 mmol per gram was used in order to ensure a high level of protection. Prior to this modification, the wood was extracted for 24 h in a Soxhlet apparatus with a solvent mixture comprising toluene/acetone/methanol 4:1:1 [88]. The thus pretreated wood subsequently was oven-dried at 105 °C for 24 h and then stored in a laboratory desiccator. In detail, a wood veneer chip of 0.129 g (0.90 mmol of accessible wood-OH) was reacted under nitrogen atmosphere with 0.304 g (1.34 mmol) 1*H*-benzotriazol-1-yl(phenyl)methanone-α-^13^C in the presence of 0.011 g (0.09 mmol) 4-N,N-dimethylaminopyridine (4-DMAP) and 0.25 mL (1.80 mmol) triethylamine in 20 mL of anhyd. DMF at 120 °C. Subsequently, the mixture was allowed to cool to r.t. and the modified wood was transferred to a filter. This product was then washed successively with THF (50 mL), chloroform (50 mL), and diethyl ether (50 mL). Afterward, the wood sample was extracted again for 24 h by applying the Soxhlet apparatus with the same solvent mixture as given above. Finally, the modified wood was dried at 105 °C for 24 h and then cooled down and stored in a desiccator. The mass increase was 15.3% WPG, equal to 1.46 mmol/g QCO (quantity of covalently bonded organomaterial) [4]. In the case of the fluorine-derived precursor, a single wood veneer chip of 0.146 g (1.02 mmol of accessible wood-OH) was reacted with 0.367 g (1.02 mmol) 1*H*-benzotriazol-1-yl[3,5-bis(trifluoromethyl)phenyl]methanone in the presence of 0.013 g (0.10 mmol) 4-DMAP and 0.28 mL (2.04 mmol) triethylamine in 20 mL of anhyd. DMF at 70 °C. After workup as described above, the mass increase was determined to be 17.9% weight percentage gain (WPG), which in this particular case was equal to 0.75 mmol/g QCO.

### 2.6. Wood Sample Preparation for Solution State NMR 

The modified wood samples were made from Scots pine (*Pinus sylvestris* L.) sapwood veneer pieces of approx. dimensions 25 × 15 × 0.6 mm^3^. A sliced piece of approx. 30 mg was transferred into a 2 mL Eppendorf vessel and was gently milled by means of two 7 mm stainless steel milling balls in a vibrational mill (MM400, Retsch, Haan, Germany) for 15 min at 30 Hz vibration frequency. Afterward, an amount of about 2–3 mg of the resulting modified wood dust was transferred into a 5 mm NMR tube (Wilmad 535-PP-7) in order to be dissolved. After the addition of about 30 mg of dried lithium chloride and about 0.6 mL of DMSO-*d_6_*, the sample was heated for 60 min at 140 °C and shaken from time to time until an appropriate solution was obtained. In contrast to significantly higher-concentrated ‘native’ cell wall NMR samples known from literature (e.g., from Mansfield and Ralph’s group [42]), in the present case under careful visual inspection, this was a true solution rather than a gel or even a slurry. For DOSY measurements, an aliquot of about 170 µL was transferred to a corresponding 3 mm high-quality NMR tube.

### 2.7. Solution State 1D and 2D Correlation NMR Spectroscopy

All NMR measurements were conducted at room temperature. Proton NMR was performed as a standard experiment (Bruker pulse program zg30) with 64 transients. For one-dimensional carbon spectra, a DEPTQ experiment was used in order to obtain sufficient signal intensity (Bruker pulse program deptqgpsp.2, 24 k transients, pulse repetition delay of 2 s). The gradient-selected ^1^H,^13^C-HMBC with low-pass filter was taken from the standard Bruker library (pp: hmbcetgpl2nd), whereas the band-selective counterpart was a constant-time version also provided by Bruker (pp: shmbcctetgpl2nd). Unless stated otherwise, all 2D correlation spectra were non-uniformly sampled with 10% NUS data. Further experimental details for 1D and 2D NMR measurements as well as selected spectra are given in the Supplementary Materials.

### 2.8. DOSY Spectroscopy 

The applied pulse sequence was the DOSY Oneshot experiment [25] performed as a pseudo-2D measurement consisting of usually 16 single 1D-spectra with squared increase of the gradient field strength. The latter was stepped up from 10 to 80%. Depending on the individual diffusion rate, the inter-gradient delay or diffusion time Δ (pulse program parameter d20) was set in a range from 0.1 s for small reference compounds to 1.0 s for the biomacromolecules. Furthermore, the corresponding length of the bipolar, magnetically opposed gradient pulse (p30; δ/2) was adjusted from 1.10 ms up to 4.25 ms [19,89]. The DOSY data were analyzed with the so-called DOSYToolbox developed by Nilsson [90] in order to calculate diffusion coefficients from the series of gradually attenuated one-dimensional spectra. Thereby, collected FIDs of each 1D-spectrum of this series were treated with an appropriate line broadening factor in the exponential multiplication step. This LB was set to 1.0 Hz in the case of small molecules or up to 5.0 Hz in the case of the macromolecular wood analytes, respectively. As no correction was included for D values taking into account the higher viscosity of the DMSO-*d_6_*/lithium chloride solvent system, we went back to the comparative method of Cabrita and Berger [91] with tetramethylsilane (TMS) as the reference also for diffusion studies in addition to its common use for shift scale referencing in the case of the NMR nuclei ^1^H, ^13^C, and ^29^Si, respectively.

## 3. Results and Discussion

### 3.1. Wood Modification: Chemical Background and Precursor Synthesis

**Scheme 1:**
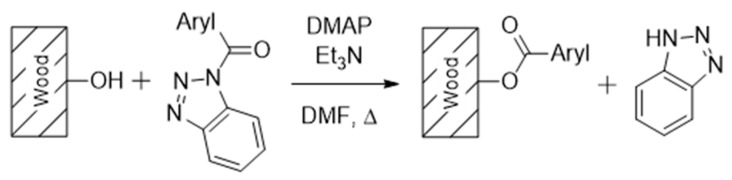
Wood esterification by means of 1*H*-benzotriazolyl activated benzoic acids.

The wood modification based on 1*H*-benzotriazolyl activation of an appropriate modifying reagent has recently been published by the Kaufmann group and is given in Scheme 1 [3,4,5,6,7,8,9].

**Scheme 2:**
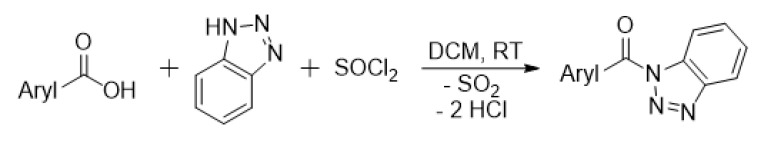
Activation of (substituted) benzoic acids to obtain appropriate wood modification reagents (Aryl = phenyl or 3,5-bistrifluoromethyl-phenyl).

The previous activation step was accomplished upon reaction of the corresponding organic acid with 1*H*-benzotriazole in dry DCM and thionyl chloride. The benzoic acid derivative (Aryl = Ph) was obtained in 85% chemical yield, whereas the fluorinated counterpart (Aryl = 3,5-bistrifluoromethyl-phenyl) was synthesized in 98% yield (Scheme 2).

**Figure 1:**
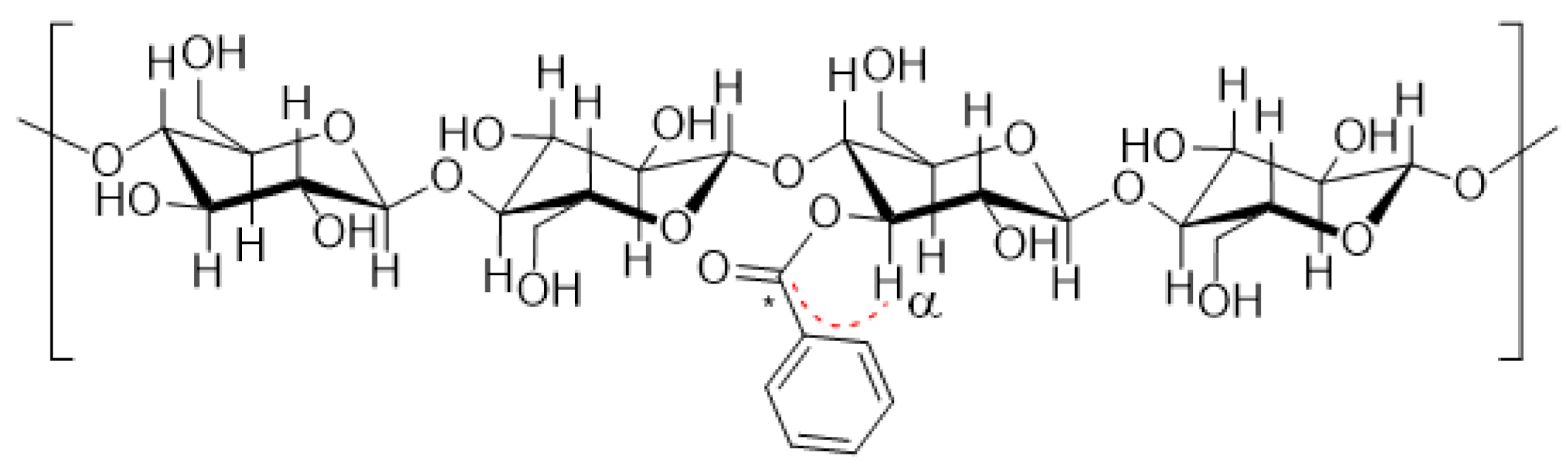
Chemical modification and corresponding NMR proof of the covalent character of such modifications graphically exemplified within a cellulosic part of wood. The ^1^H,^13^C-HMBC confirmed ^3^*J*_C,H_ spin–spin coupling cross signal (see text) is marked with a red dotted line. Additional cross peaks attributed to corresponding modification of lignin parts in addition to cellulose and hemicellulose regions are given as original NMR cross peaks in Figure 4.

The chemical modification of wood with this phenyl derivative was then achieved at 120 °C by applying 1.5 equivalents of the activated α-^13^C-benzoic acid. The molar ratio was based upon 7 mmol of accessible hydroxyl groups per grams of wood according to the literature [92]. Scots pine (*Pinus sylvestris* L.) sapwood veneer was used as the wood material. The resulting mass increase was determined to be 15.3% WPG, which is equal to a QCO value of 1.46 mmol reagent per gram of wood. Even on application of the quite potent LiCl/DMSO-*d_6_* mixture for the preparation of the NMR sample, the de facto dissolvable amount of the entire wood was still very low. Therefore, a ^13^C-labeling of the benzoate carbonyl C*-atom of the modifying reagent was inevitable for this task. Figure 1 depicts the obtained connectivity of the precursor molecule to the cellulose- or hemicellulose-type wood structure. Spectroscopically, this is verified by a corresponding ^1^H,^13^C-HMBC cross signal that is caused by the three-bond (^3^*J*_C,H_) coupling from the proton in the α-position of the former wood hydroxyl group to the carbonyl C-atom of the modifying benzoate.

### 3.2. Solution State NMR of Modified Wood Samples

**Figure 2:**
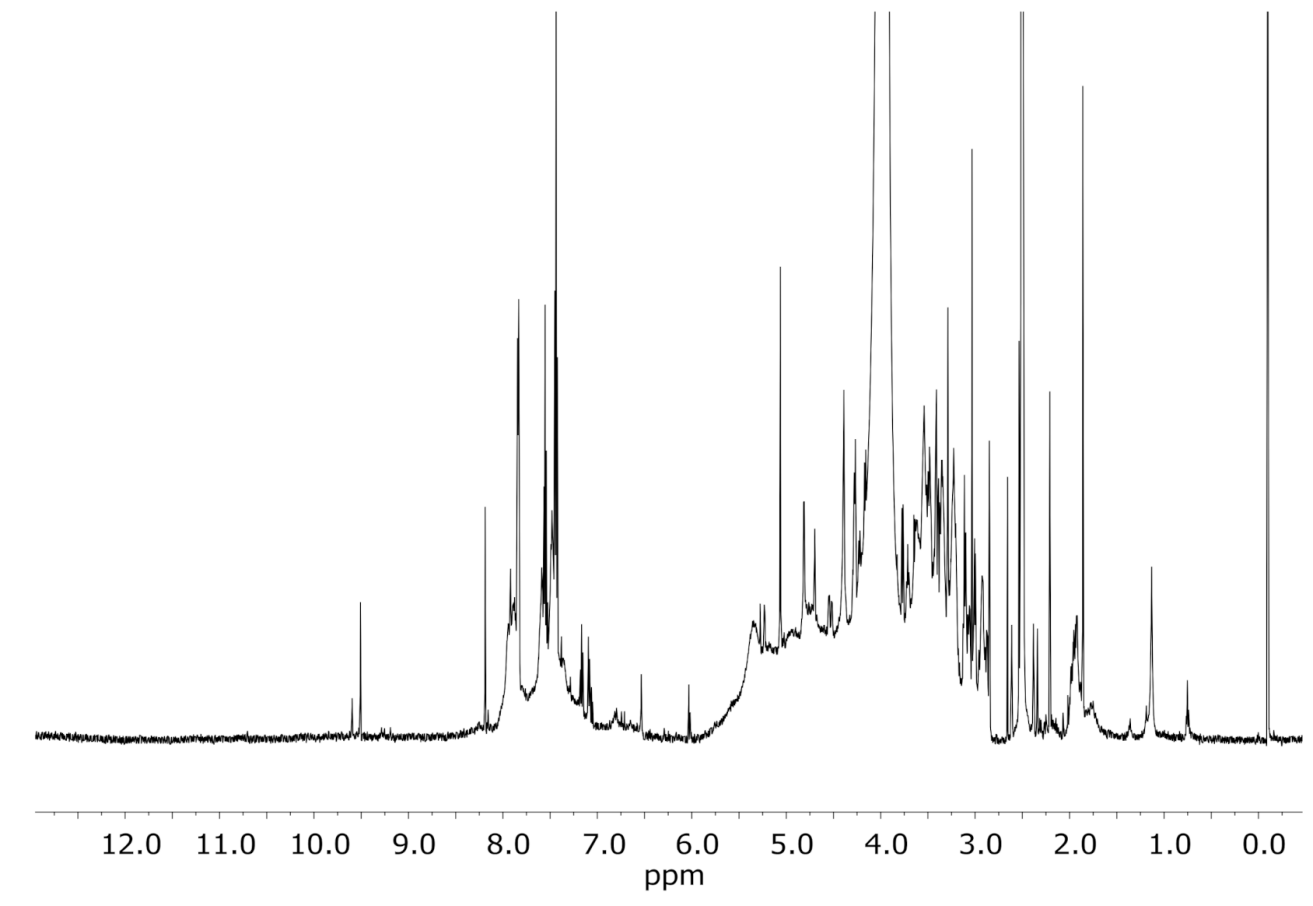
The 600 MHz proton NMR spectrum of benzoylated wood in DMSO*-d_6_*/LiCl, 64 transients.

**Figure 3:**
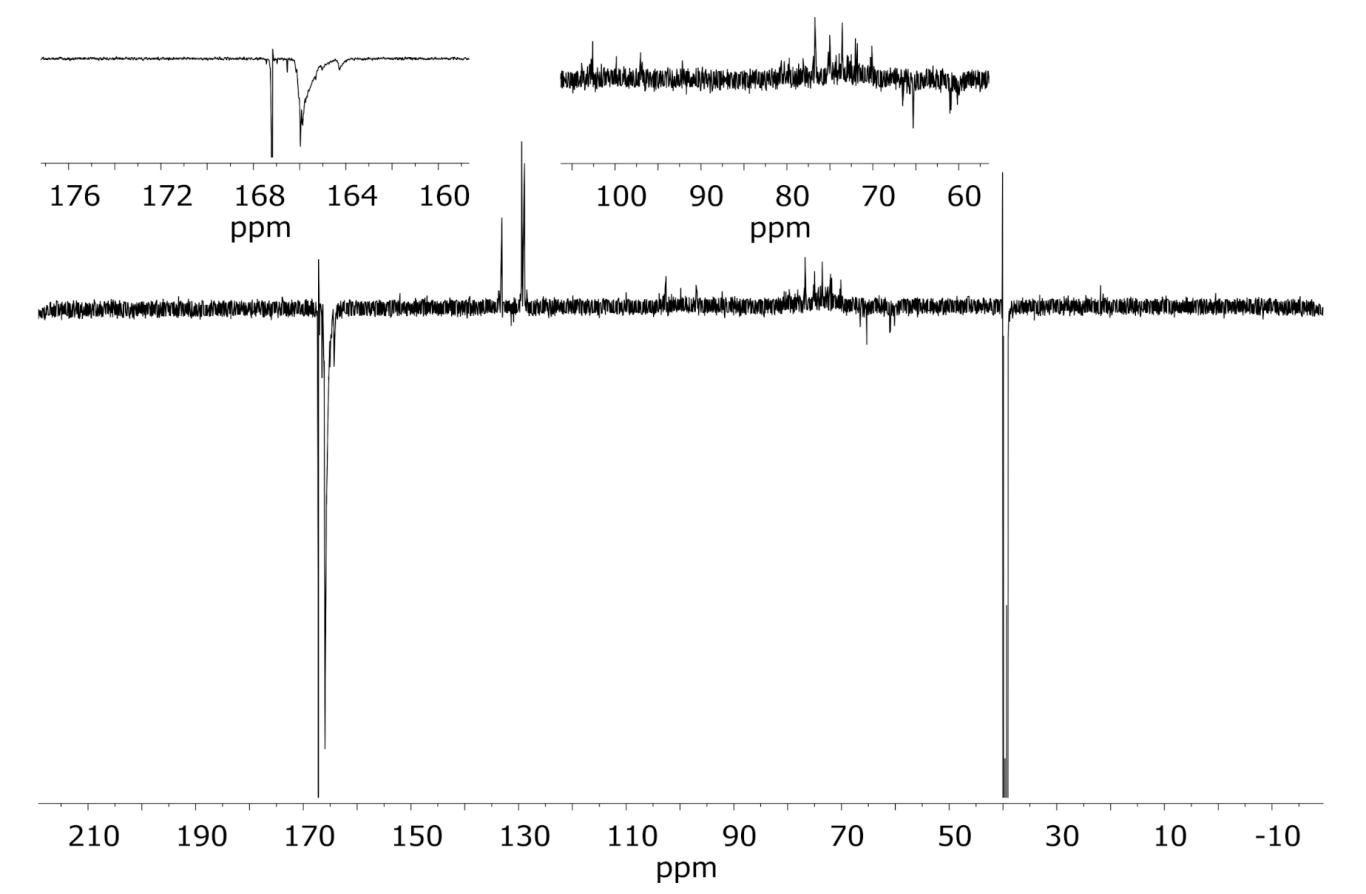
DEPTQ carbon NMR spectrum of benzoylated wood in DMSO*-d_6_*/LiCl, 24k transients.

In Figure 2 and Figure 3, the initially obtained proton and carbon NMR (DEPTQ) [93,94] spectra of the thus dissolved modified wood are depicted. The ^1^H NMR spectrum gives an impressive signal-to-noise ratio providing aromatic signals from lignin as well as carbohydrate resonances that can be attributed to (hemi)cellulose regions of wood. From the appearance of these two signal regions, it was confirmed that not only structural parts, but the entire wood material was dissolved as desired. However, the carbon spectrum still suffered from the low concentration of the analyte, except for the ^13^C-labeled carbonyl group at about 170 ppm, which in addition to the DMSO-signal at 40 ppm led to an outstanding s/n ratio as expected. The enlarged region of the carbonyl signals showed that there were at least four aromatic ester species: sharp signals of different intensities and probably two broad signals with high and low intensity (from higher to lower ppm values). Thereby, the sharp signals reflect non-polymeric structures, consequently not attached to wood, but free species. In contrast to this, the broadness of the two other signals undoubtedly points to the chemical modification of the wood. In detail, a covalent attachment should cause a number of similar resonances that are due to structurally related but in detail nonidentical wood substructures where the benzoate is attached. The second region that is depicted as a zoom in Figure 3 shows the carbohydrate resonances from about 60 to 110 ppm, hence the cellulose and hemicellulose part of the dissolved wood: a fact that was also reflected by the corresponding aliphatic region of the proton NMR spectrum.

**Figure 4:**
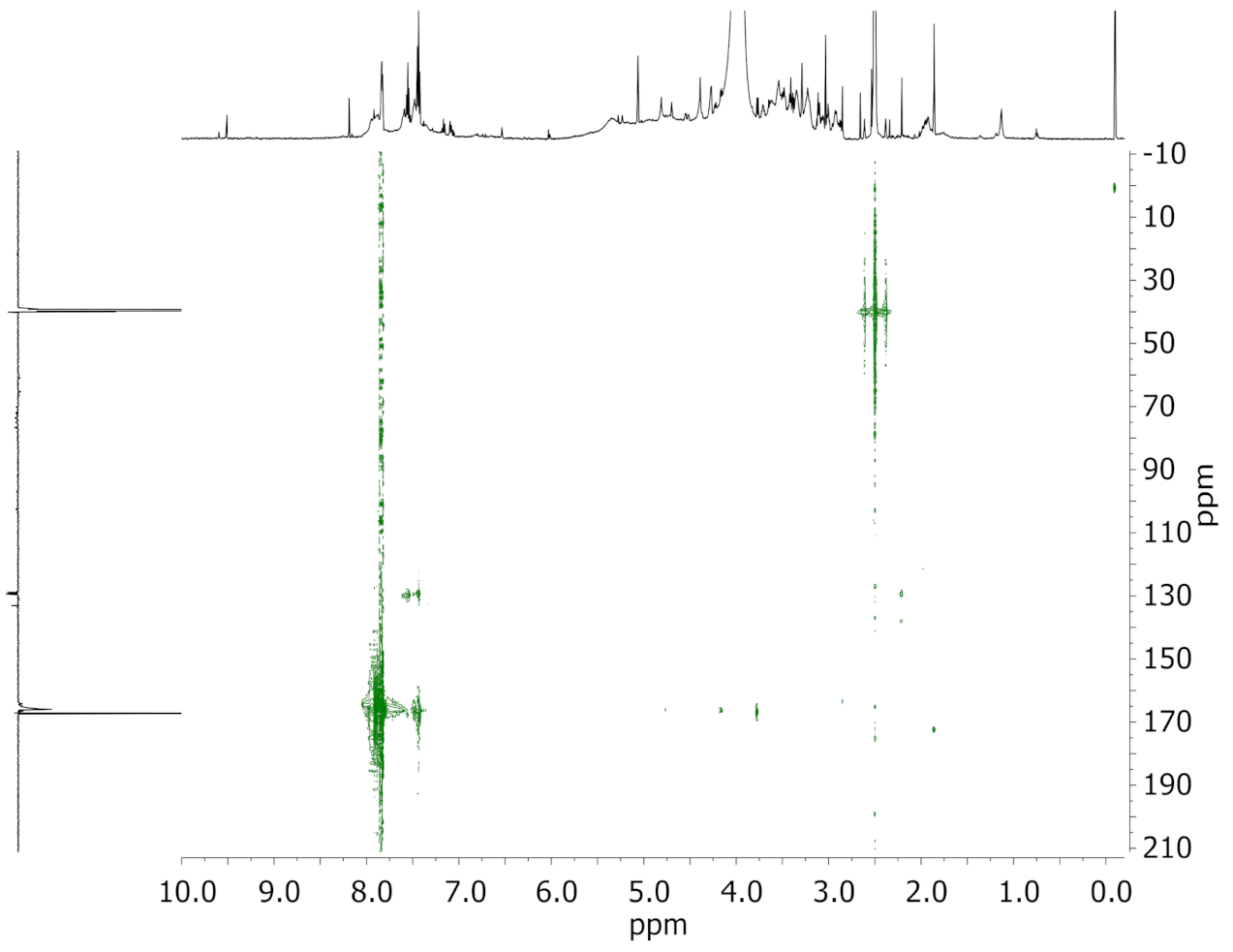
Gradient-selected ^1^H,^13^C-HMBC NMR spectrum of benzoylated wood in DMSO-*d_6_*/LiCl (non-uniformly recorded with 10% NUS data).

While the two preceding spectra could be recorded within minutes (^1^H NMR, 64 scans) or within several hours in the case of the carbon spectrum (24 k scans), the measurement of a ^1^H,^13^C-HMBC required 2800 scans per increment. To reach adequate resolution in an acceptable period of time, measurement was carried out by non-uniform sampling (NUS), recording only 10% of the regular NMR data within four days and 18 h for 80 measured increments. The subsequent reconstruction of the non-uniformly sampled data was accomplished with the iterative soft thresholding algorithm tool (hmsIST) of the Wagner group [16]. Figure 4 shows the thus processed HMBC spectrum. In particular, the cross signals at the 3.78, 4.17, and 4.76 ppm proton chemical shift were of interest as they confirm covalent connectivity over three bonds to the introduced carbonyl carbon atoms of the benzoate.

Upon closer inspection, the correlation of the proton signal around 3.8 ppm was to a small, but relatively sharp carbon signal at 166.6 ppm. The small half width of this resonance as well as the proton and carbon shift values indicate a small amount of non-bonded α-^13^C-methyl benzoate. This compound may be built up as a methyl ester upon the initial heating during sample preparation. It is well known that methanol (so-called wood spirit) is released from wood at elevated temperatures. This alcohol is then able to undergo a transesterification with wood-benzoate positions, resulting in the formation of methyl benzoate, or traces of methanol trap a little bit of the highly reactive benzoate precursor. The other two signals above 4 ppm proton shift both have a correlation to more shielded and broad carbonyl signals for which a much higher resolution in F1 direction (^13^C) was necessary. In order to manage this, a band-selective ^1^H,^13^C-HMBC [95,96] was acquired around the carbonyl region, again using 10% NUS data. For a carbon band width of 8.5 ppm, this took 3 k scans per every of the 40 increments recorded, resulting in a total measurement time of 68 h. 

**Figure 5:**
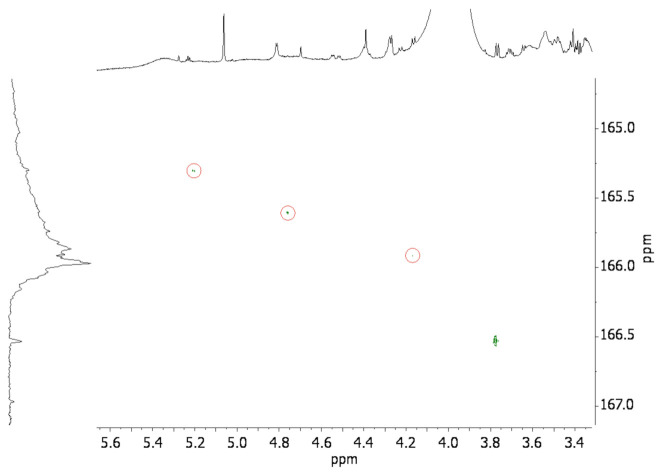
Band-selective ^1^H,^13^C-HMBC NMR spectrum of benzoylated wood in DMSO-*d_6_*/LiCl (non-uniformly recorded with 10% NUS data), weak cross-signals are marked with circles.

This 2D correlation spectrum (Figure 5) impressively depicts the resolution enhancement that allows for at least partial structural assignment: even though the cross-signals at 4.17 (correlated to 165.9 ppm carbon shift) and 4.76 ppm proton shift (^13^C at 165.6 ppm), respectively, are attributable to (hemi)cellulose substructures or aliphatic C_3_-fragments of lignin, an additional very instructive cross-peak was detected at 5.20 ppm (^13^C: 165.3 ppm). This downfield proton shift value unambiguously indicates a carbohydrate resonance, a fact that not only proves the covalent character of the attachment, but furthermore provides information about the actual position within the wood constituents, in particular at cellulose or hemicellulose structural parts. In detail, for α-D(1-4) linked glucopyranose (i.e., the cellulose backbone) the above-mentioned value of the 5.20 ppm proton shift relates to the 3-position of glucose units as depicted in Figure 1. These findings have been verified by applying NMR shift predictions with the ACD/C+H Predictors 2020 [97] and with analogous NMR experiments that were performed with cellulose triacetate.

### 3.3. Comparison to Corresponding Spectra of Cellulose Triacetate as a Similarly Esterified Model Compound 

To obtain more insight into the regiochemical aspects of the unquestionable covalent wood modification as confirmed by the solution state NMR experiments, the corresponding NMR spectra were recorded with cellulose triacetate as the analyte (see Supplementary Materials). The HMBC revealed that only the protons in the 2- and 3-position of cellulose units have a long-range coupling correlation to the ester carbonyl carbon atom. This assignment is in agreement with standard NMR literature [98] and with NMR shift predictions [97].

### 3.4. Diffusion Ordered NMR Spectroscopy (DOSY)

Making use of any kind of proton spectroscopy in wood chemistry always leads to a strong overlap. Hence, for our purpose, we decided to preferentially use the ^19^F nucleus, which offers a similar sensitivity due to its natural abundance of 100% and its high gyromagnetic ratio γ. Some early papers are known, which deal with this nucleus, especially in lignin analysis (e.g., by Barelle [99,100] and by Argyropoulos and co-workers [101,102]). The conventional Oneshot DOSY experiment of Morris and co-workers [25] was the perfect choice as with our trifluoromethyl-substituted precursor and thus modified wood, the full bandwidth of ^19^F, which was taken into account by a more recent pulse program developed by the same group [28], was not required. In each of the recorded ^19^F-DOSY spectra of the precursor and the fluorinated biomacromolecule, a single ^19^F signal was expected. 

Prior to these DOSY experiments, regular 1D spectra of both nuclei of the thus modified wood were obtained (see Supplementary Materials). As expected, in the ^1^H spectrum (64 transients) a hump-like background from about 3 to 6 ppm chemical shift (carbohydrate region) appeared; in addition, there was a less intensive aromatic signal of the lignin constituents around 7 ppm. Similar to the appearance of the proton signals in Figure 2, additional polymer-based signals (smaller, but still overlapping) were found on top of these humps. Due to the limited signal-to-noise ratio, the largest peaks in the proton spectrum were caused by residual solvent protons (i.e., partly undeuterated forms of deuterodimethyl sulfoxide) and by a certain water content that is originally present in commercially available DMSO-*d_6_*; it is even provided in ampoules. It was noted that traces of water in the DMSO-*d_6_*/LiCl system appeared at about the 3.9 ppm proton chemical shift, which is in contrast to the usual 3.3 ppm resonance of water in pure DMSO-*d_6_*. As probable solvent suppression would not solve the issue of signal overlap in the proton case, we were prompted to prefer the ^19^F-based detection upon subsequent DOSY measurements. Prior to this, we recorded the necessary one-dimensional fluorine NMR spectrum, which was due to the chemical and magnetic equivalence of all fluorine atoms within the bis(trifluoromethyl)phenyl derivative, and showed a single signal (broadened) of the covalently attached fluorine-derived benzoate at around −61.4 ppm and an additional sharp signal of residual, non-bonded free acid that originated from small amounts of partly hydrolyzed fluorine-derived precursor (see Supplementary Materials). In this context, it clearly points out that forced treatment of covalently modified wood veneer chips under harsh hydrolysis conditions (in detail, boiling water applied for 24 h) did not result in any weight loss that could have been caused by ester cleavage.

With these 1D spectra in hands that were obtained within minutes despite the low sample concentration, we then aimed at DOSY spectroscopy as a less time-consuming alternative to the above discussed HMBC approach. This DOSY technique is an analysis tool for virtual separation of molecules based on their (different) diffusion rates. It dates back to 1992 when Morris and Johnson Jr. published a data processing method that allowed for the calculation as well as appropriate presentation of diffusion data as a two-dimensional DOSY plot of chemical shifts and corresponding diffusion rates, respectively [20]. The basic DOSY data are obtained from a series of successively attenuated one-dimensional spectra of a sensitive nucleus, in most cases ^1^H. In detail, from one 1D NMR spectrum to another, each single spectrum is recorded by applying a varied pair of pulses provided by a gradient coil to at first encode and then decode diffusing molecules. The inter-gradient delay was the diffusion time (usually about some hundred milliseconds) and the gradient length was set to approx. values between 1.0 and 4.0 ms. From the observed mono-exponential decay of the individual signal intensities *I*, the diffusion rates *D* were calculated upon an appropriate curve fitting by means of the equation introduced by Stejskal and Tanner [103]:

(1) $$I=I_{0}e^{-D\gamma^{2}G^{2}\delta^{2}\frac{\Delta ࢤ\delta }{3}}$$

In addition to the already mentioned parameters (i.e., gradient length and diffusion time as well as the z-gradient strength *G*), the *I*_0_ in this formula reflects the signal intensity without diffusion. 

**Figure 6:**
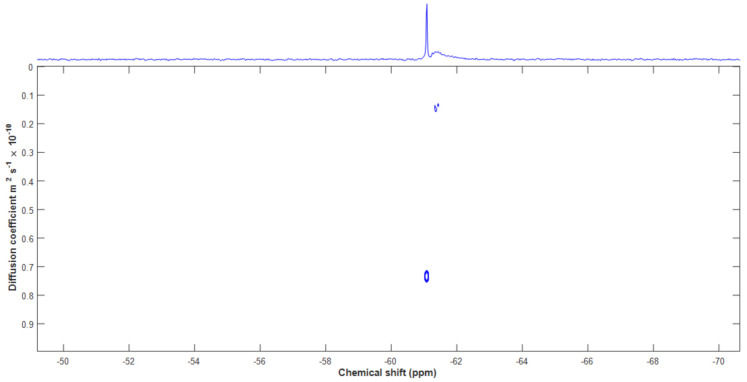
^19^F-detected DOSY of chemically modified wood dissolved in DMSO*-d_6_*/LiCl with a diffusion rate of about 1.2∙10^−11^ m^2^∙s^−1^.

In our case, these measurements were performed with the one-shot pulse sequence published by the group of Morris [25]. Subsequent calculations of the diffusion coefficients were accomplished with version 2.5 of the DOSYToolbox introduced by Nilsson [90]. In detail, the ^19^F-DOSY measurement of the bis(trifluoromethyl)benzoyl-derived wood was performed in 16 squared steps from 10 to 80% gradient strength with a diffusion time of 750 ms and a gradient length of 4 ms. It unambiguously indicated the actual esterification and thus the covalent type of strong bonding of the precursor to the wood veneer chip by an extraordinary low diffusion coefficient of about 1.2∙10^−11^ m^2^∙s^−1^. This result was easily extracted from the DOSYToolbox and was clearly shown in the corresponding DOSY plot (see Figure 6).

**Figure 7:**
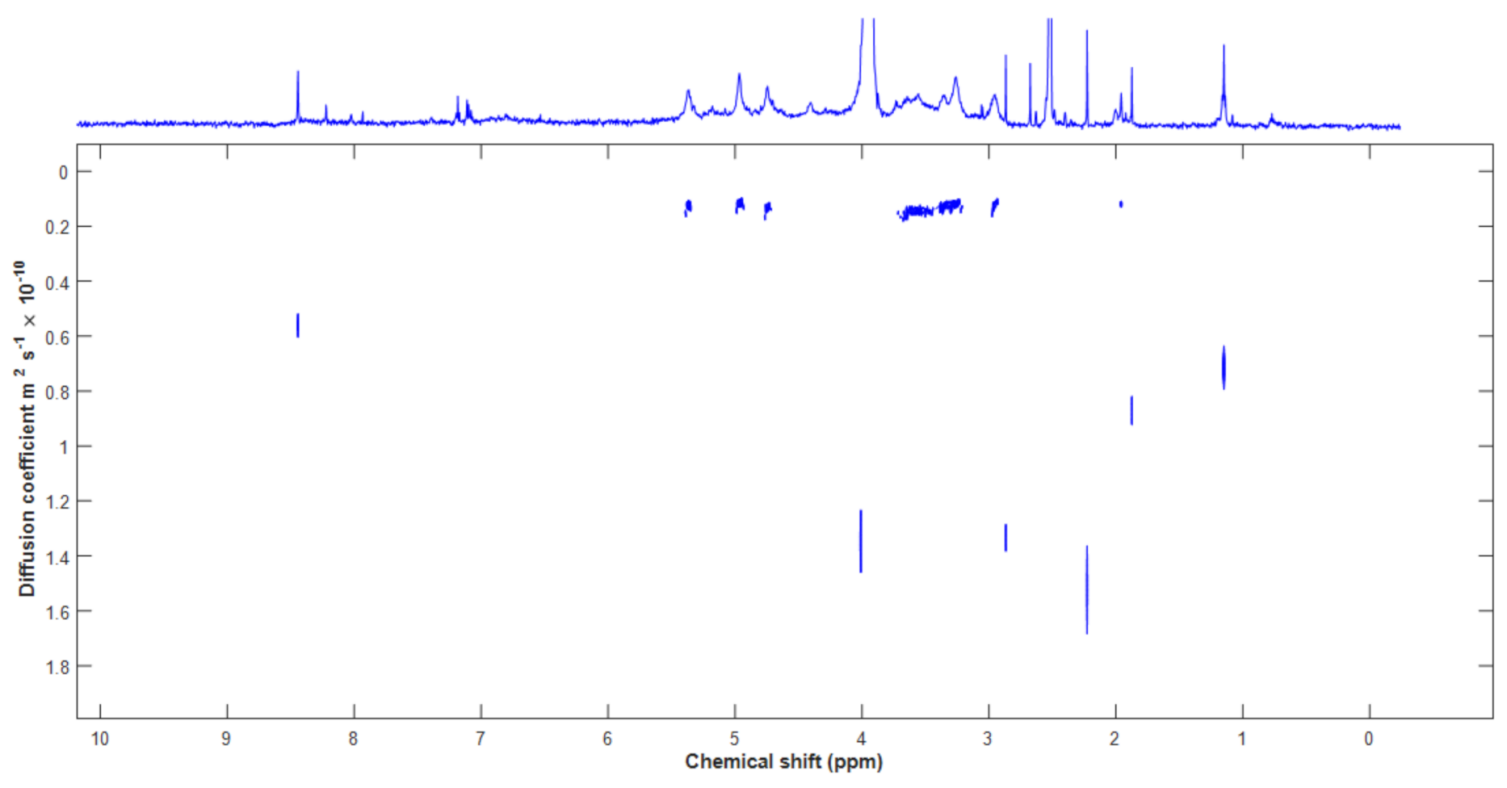
Crowded ^1^H-detected DOSY of chemically modified wood dissolved in DMSO*-d_6_*/LiCl corresponding to the ^19^F-DOSY counterpart depicted in Figure 6.

An additional proton-detected diffusion NMR confirmed the above-mentioned D value of the chemically modified wood, although with less clarity as expected (see Figure 7).

**Figure 8:**
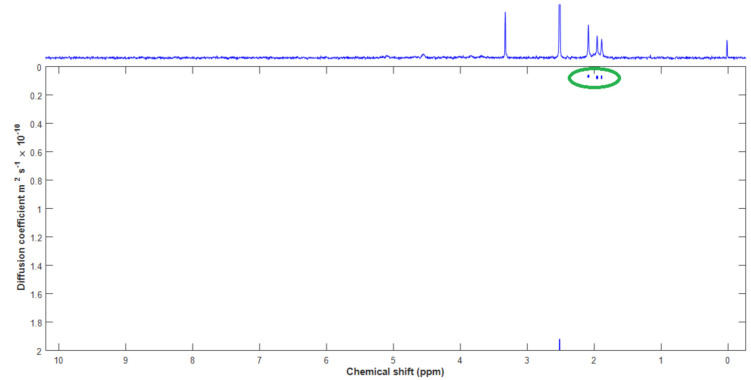
^1^H-detected DOSY of commercially available cellulose triacetate dissolved in DMSO*-d_6_* with a diffusion rate of about 0.8∙10^−11^ m^2^∙s^−1^ (D value scale as before for comparison).

As a model compound, commercially available cellulose triacetate was measured in the same DMSO-*d_6_*/LiCl solvent system applying an intergradient delay of 1.0 s and a gradient length of 4.25 ms (also 16 squared steps from 10 to 80% gradient strength). The diffusion rate D that results from this ^1^H-DOSY (see Figure 8) was about 0.3∙10^−11^ m^2^∙s^−1^, whereas the corresponding ^1^H-DOSY in pure DMSO-*d_6_* gave a *D* value of about 0.8∙10^−11^ m^2^∙s^−1^ (refer to S12 in the Supporting Information ). The significant differences in *D* values caused by DMSO-*d_6_*/LiCl and pure DMSO, respectively, were in accordance with the dependency of *D* from the viscosity of the solution as taken into consideration in the common Stokes–Einstein equation:

(2) $$D=\frac{k\;T}{6\;\pi \;\eta \;r}$$

However, it should have been considered that the actual shape of the polymeric compounds is by far not spherical, but this is commonly neglected upon calculation of the diffusion rates.

In analogy to the measurements of the above described DOSY spectra, tetramethylsilane (TMS) was measured in order to allow for a kind of diffusion rate referencing that was introduced by Cabrita and Berger [91]. The according D value for TMS in the DMSO-*d_6_*/LiCl solvent system was found to be (2.33 ± 0.04)∙10^−10^ m^2^∙s^−1^, whereas the D value in DMSO-*d_6_* with traces of H_2_O due to the hygroscopicity of this solvent was (5.85 ± 0.02)∙10^−10^ m^2^∙s^−1^. Last, but not least, independent DOSY measurements of the small precursor molecules resulted in D values of about 1.2∙10^−10^ m^2^∙s^−1^ for the methyl ester and about 0.7∙10^−10^ m^2^∙s^−1^ for the free acid (probably as a dimer) in DMSO-*d_6_*/LiCl (refer to Supplementary Materials) and about 4∙10^−10^ m^2^∙s^−1^ in pure DMSO-*d_6_*. Again, the large diffusion coefficients for the small reference compounds were as expected in contrast to the slowly diffusing precursor after its covalent attachment to the biomacromolecular wood as depicted in Figure 6 and Figure 7.

An overview of the diffusion coefficients of chemically modified wood, applied precursors, and reference compounds is given in Table 1. More details on the NMR measurement parameters are given in the Supplementary Materials.

## 4. Conclusions

Contemporary chemical wood modification procedures, especially based upon esterification of wood hydroxyl groups applying (1*H*-benzotriazolyl)-activated benzoic acids, lead to a covalent attachment of the functionalizing reagent. These findings were achieved by means of solution state 2D NMR spectroscopy at room temperature by conducting adapted heteronuclear multiple bond correlation 2D NMR experiments (i.e., standard and band-selective ^1^H,^13^C-HMBC, respectively). These 2D NMR techniques were performed with time efficient fractional data recording and subsequent reconstruction (i.e., with non-uniform sampling of only 10% of regular uniformly achieved NMR data), so that 90% of the usually necessary measurement time was saved. In the HMBC spectra, specific cross-signals were obtained that arose from spin–spin coupling over three electron pair bonds, especially from the ester carbonyl carbon atom to proton atoms in the α-position of the (then esterified) wood hydroxyl groups. Even though the necessary measurement time was decreased to the tenth part of the usual recording time, an even more rapid test that depicts successful attachment to the polymeric backbone upon this type of chemical modification of wood was achieved by applying an up-to-date variant of diffusion-ordered spectroscopy (one-shot version of a DOSY pulse sequence) that was developed by Morris and co-workers [25]. The appropriate wood solution was obtained by a mild and non-destructive method.

## Figures and Tables

**Table 1:** Diffusion coefficients of chemically modified wood, suitable precursors, and reference compounds.

Entry	Compound	Solvent	Diffusion Coefficient D [10^−10^ m^2^∙s^−1^]
1	3,5-(CF_3_)_2_C_6_H_3_-COO-wood ^a^	LiCl/DMSO-*d_6_*	0.120 ± 0.012 ^b,d^
2			0.121 ± 0.005 ^c^
3	cellulose triacetate	LiCl/DMSO-*d_6_*	0.028 ± 0.002 ^c^
4		DMSO-*d_6_*	0.081 ± 0.002 ^c^
5	tetramethylsilane (TMS)	LiCl/DMSO-*d_6_*	2.33 ± 0.04 ^c^
6		DMSO-*d_6_*	5.85 ± 0.02 ^c^
7	3,5-(CF_3_)_2_C_6_H_3_-COOH	LiCl/DMSO-*d_6_*	0.71 ± 0.00(6) ^b^
8		DMSO-*d_6_*	3.59 ± 0.04 ^b^
9	3,5-(CF_3_)_2_C_6_H_3_-COOMe	LiCl/DMSO-*d_6_*	1.22 ± 0.06 ^b^
10		DMSO-*d_6_*	4.45 ± 0.10 ^b^

^a^ about 1.7 mg of chemically modified wood (as a dust resulting from mild ball-milling) in 170 µL NMR solvent, ^b 19^F-DOSY, ^c 1^H-DOSY, ^d^ additional free acid gave a *D* value of about 0.79∙10^10^ m^2^∙s^−1^.

## Data Availability

Not applicable.

## Supplementary Materials

The following are available online at https://www.mdpi.com/article/10.3390/polym13132164/s1. Detailed NMR parameters, comprehensive sets of NMR spectra of cellulose triacetate as the model compound of the activated functionalized precursor 1*H*-benzotriazol-1-yl][3,5-bis(trifluoromethyl) phenyl]methanone of the modified wood. DOSY spectra of the free acid and its methyl ester, and detailed parameters of all DOSY spectra are available online.

## References

[1] Hill C.A.S. (2006). Wood Modification. Chemical, Thermal and Other Processes.

[2] Rowell R.M. (2013). Handbook of Wood Chemistry and Wood Composites.

[3] Namyslo J.C., Kaufmann D.E. (2009). Chemical improvement of surfaces. Part 1: Novel functional modification of wood with covalently bound organoboron compounds. Holzforschung.

[4] Drafz M.H.H., Dahle S., Maus-Friedrichs W., Namyslo J.C., Kaufmann D.E. (2012). Chemical improvement of surfaces. Part 2: Permanent hydrophobization of wood by covalently bonded fluoroorganyl substituents. Holzforschung.

[5] Namyslo J.C., Kaufmann D.E., Mai C., Militz H. (2015). Chemical Improvement of surfaces. Part 3: Covalent modification of Scots pine sapwood with substituted benzoates providing resistance to *Aureobasidium pullulans* staining fungi. Holzforschung.

[6] Kaldun C., Dahle S., Maus-Friedrichs W., Namyslo J.C., Kaufmann D.E. (2016). Chemical improvement of surfaces. Part 4: Significantly enhanced hydrophobicity of wood by covalent modification with *p*-silyl-functionalized benzoates. Holzforschung.

[7] Kaldun C., Söftje M., Namyslo J.C., Kaufmann D.E. (2020). Chemical improvement of surfaces. Part 5: Surfactants as structural lead for wood hydrophobization—Covalent modification with *p*-alkylated benzoates. Holzforschung.

[8] Ehrhardt C., Tapken M., Namyslo J.C., Kaufmann D.E. (2021). Chemical improvement of surfaces. Part 6: Enhanced flame retardancy of Scots pine sapwood by covalent modification with phosphorus and boron functionalized benzoates. Holzforschung.

[9] Söftje M., Acker S., Plarre R., Namyslo J.C., Kaufmann D.E. (2020). Chemical improvement of surfaces. Part 7: Novel nicotinoid structures for covalent modification of wood: An environmentally friendly way for its protection against insects. RSC Adv..

[10] Hill C.A.S., Jones D. (1999). Dimensional Changes in corsican pine sapwood due to chemical modification with linear chain anhydrides. Holzforschung.

[11] Wienhaus O., Niemz P., Fabian J. (1988). Untersuchungen zur Holzartendifferenzierung mit Hilfe der Infrarotspektroskopie. Teil 1. Holzforsch. Holzverwert..

[12] Niemz P., Wienhaus O., Schaarschmidt K., Ramin R. (1989). Untersuchungen zur Holzartendifferenzierung mit Hilfe der Infrarotspektroskopie, Teil 2. Holzforsch. Holzverwert..

[13] Zollfrank C., Wegener G. (2002). FTIR microscopy and ultrastructural investigation of silylated solid wood. Holzforschung.

[14] Tjeerdsma B.F., Militz H. (2005). Chemical changes in hydrothermal treated wood: FTIR analysis of combined hydrothermal and dry heat-treated wood. Holz Roh Werkst..

[15] Stefke B., Windeisen E., Schwanninger M., Hinterstoisser B. (2008). Determination of the weight percentage gain and of the acetyl group content of acetylated wood by means of different infrared spectroscopic methods. Anal. Chem..

[16] Hyberts S.G., Milbradt A.G., Wagner A.B., Arthanari H., Wagner G. (2012). Application of iterative soft thresholding for fast reconstruction of NMR data non-uniformly sampled with multidimensional Poisson Gap scheduling. J. Biomol. NMR.

[17] Hyberts S.G., Arthanari H., Robson S.A., Wagner G. (2014). Perspectives in magnetic resonance: NMR in the Post-FFT Era. J. Magn. Reson..

[18] Pedersen C.P., Prestel A., Teilum K. (2021). Software for reconstruction of nonuniformly sampled NMR data. Magn. Reson. Chem..

[19] Stilbs P. (1981). Molecular Self-diffusion coefficients in fourier transform nuclear magnetic resonance spectrometric analysis of complex mixtures. Anal. Chem..

[20] Morris K.F., Johnson C.S. (1992). Diffusion-ordered two-dimensional nuclear magnetic resonance spectroscopy. J. Am. Chem. Soc..

[21] Price W.S. (1997). Pulsed-field gradient nuclear magnetic resonance as a tool for studying translational diffusion: Part 1. Basic theory. Concepts Magn. Reson..

[22] Pelta M.D., Barjat H., Morris G.A., Davis A.L., Hammond S.J. (1998). Pulse sequences for high-resolution diffusion-ordered spectroscopy (HR-DOSY). Magn. Reson. Chem..

[23] Johnson C.S. (1999). Diffusion ordered nuclear magnetic resonance spectroscopy: Principles and applications. Prog. Nucl. Magn. Reson. Spectrosc..

[24] Morris G.A., Grant D.M., Harris R.K. (2002). Encyclopedia of NMR.

[25] Pelta M.D., Morris G.A., Stchedroff M.J., Hammond S.J. (2002). A one-shot sequence for high-resolution diffusion-ordered spectroscopy. Magn. Reson. Chem..

[26] Brand T., Cabrita E.J., Berger S. (2005). Intermolecular interaction as investigated by NOE and diffusion studies. Prog. Nucl. Magn. Reson. Spectrosc..

[27] Macchioni A., Ciancaleoni G., Zuccaccia C., Zuccaccia D. (2012). Diffusion Ordered NMR Spectroscopy (DOSY). Supramolecular Chemistry: From Molecules to Nanomaterials.

[28] Power J.E., Foroozandeh M., Moutzouri P., Adams R.W., Nilsson M., Coombes S.R., Phillips A.R., Morris G.A. (2016). Very broadband diffusion-ordered NMR spectroscopy: ^19^F DOSY. Chem. Commun..

[29] Sulaeva I., Sumerskii I., Bacher M., Zinovyev G., Henniges U., Rosenau T., Potthast A. Comparing different approaches to measure molar mass of lignin: SEC, DOSY and AsFIFFF. Proceedings of the 249th ACS National Meeting & Exposition.

[30] Montgomery J.R.D., Lancefield C.S., Miles-Barrett D.M., Ackermann K., Bode B.E., Westwood N.J., Lebl T. (2017). Fractionation and DOSY NMR as analytical tools: From model polymers to a technical lignin. ACS Omega.

[31] Cornejo A., García-Yoldi I., Alegria-Dallo I., Galilea-Gonzalo R., Hablich K., Sánchez D., Otazu E., Funcia I., Gil M.J., Martínez-Merino V. (2020). Systematic diffusion-ordered spectroscopy for the selective determination of molecular weight in real lignins and fractions arising from base-catalyzed depolymerization reaction mixtures. ACS Sustain. Chem. Eng..

[32] King A.W.T., Jalomäki J., Granström M., Argyropoulos D.S., Heikkinen S., Kilpeläinen I. (2010). A new method for rapid degree of substitution and purity determination of chloroform-soluble cellulose esters, using 31P NMR. Anal. Methods.

[33] Ohkoshi M., Kato A. (1993). Determination of substituent distribution of DMSO-soluble portion of acetylated wood meal by 13C-NMR spectroscopy. Mokuzai Gakkaishi.

[34] Ohkoshi M., Kato A., Hayashi N. (1997). 13C-NMR analysis of acetyl groups in acetylated wood I. Acetyl groups in cellulose and hemicellulose. Mokuzai Gakkaishi.

[35] Ohkoshi M., Kato A. (1997). 13C-NMR analysis of acetyl groups in acetylated wood II. Acetyl groups in lignin. Mokuzai Gakkaishi.

[36] Ohkoshi M., Kato A., Suzuki K., Hayashi N., Ishihara M. (1999). Characterization of acetylated wood decayed by brown-rot and white-rot fungi. J. Wood Sci..

[37] Moulthrop J.S., Swatloski R.P., Moyna G., Rogers R.D. (2005). High-resolution ^13^C NMR studies of cellulose and cellulose oligomers in ionic liquid solutions. Chem. Commun..

[38] Lu F., Ralph J. (2003). Non-degradative dissolution and acetylation of ball-milled plant cell walls: High-resolution solution-state NMR. Plant J..

[39] Ralph J., Lu F. (2004). Cryoprobe 3D NMR of acetylated ball-milled pine cell walls. Org. Biomol. Chem..

[40] Jiang N., Pu Y., Ragauskas A.J. (2010). Rapid determination of lignin content via direct dissolution and 1H NMR analysis of plant cell walls. ChemSusChem.

[41] Kim H., Ralph J. (2010). Solution-state 2D NMR of ball-milled plant cell wall gels in DMSO-d6/pyridine-d5. Org. Biomol. Chem..

[42] Mansfield S.D., Kim H., Lu F., Ralph J. (2012). Whole plant cell wall characterizationS using solution-state 2D NMR. Nat. Protoc..

[43] Cheng K., Sorek H., Zimmermann H., Wemmer D.E., Pauly M. (2013). Solution-state 2D NMR spectroscopy of plant cell walls enabled by a dimethylsulfoxide-d6/1-ethyl-3-methylimidazolium acetate solvent. Anal. Chem..

[44] Fukagawa N., Meshitsuka G., Ishizu A. (1991). A two-dimensional NMR study of birch milled wood lignin. J. Wood Chem. Technol..

[45] Landucci L.L., Deka G.C., Roy D.N. (1992). A 13 C NMR study of milled wood lignins from hybrid *salix* clones. Holzforschung.

[46] Li S., Lundquist K. (1994). A new method for the analysis of phenolic groups in lignins by 1H NMR spectroscopy. Nord. Pulp Pap. Res. J..

[47] Galkin S., Ämmälahti E., Kilpeläinen I., Brunow G., Hatakka A. (1997). Characterisation of Milled Wood Lignin from Reed Canary Grass (*Phalaris arundinacea*). Holzforschung.

[48] Li S., Lundquist K. (2001). Analysis of hydroxyl groups in lignins by 1H NMR spectrometry. Nord. Pulp Pap. Res. J..

[49] Heikkinen S., Toikka M.M., Karhunen P.T., Kilpeläinen I.A. (2003). Quantitative 2D HSQC (Q-HSQC) via suppression of j-dependence of polarization transfer in NMR spectroscopy: Application to wood lignin. J. Am. Chem. Soc..

[50] Holtman K.M., Chang H.-M., Kadla J.F. (2007). An NMR comparison of the whole lignin from milled wood, MWL, and REL dissolved by the DMSO/NMI procedure. J. Wood Chem. Technol..

[51] Crestini C., Melone F., Sette M., Saladino R. (2011). Milled wood lignin: A linear oligomer. Biomacromolecules.

[52] Rencoret J., del Río J.C., Gutiérrez A., Martínez Á.T., Li S., Parkas J., Lundquist K. (2012). Origin of the acetylated structures present in white birch (*Betula pendula* Roth) milled wood lignin. Wood Sci. Technol..

[53] Capanema E.A., Balakshin M.Y., Chen C.-L., Gratzl J.S., Gracz H. (2001). Structural analysis of residual and technical lignins by 1H-13C correlation 2D NMR-spectroscopy. Holzforschung.

[54] Pinto P.C., Evtuguin D.V., Pascoal Neto C., Silvestre A.J.D., Amado F.M.L. (2002). Behavior of eucalyptus globulus lignin during kraft pulping. II. Analysis by NMR, ESI/MS, AND GPC. J. Wood Chem. Technol..

[55] Ekholm F.S., Ardá A., Eklund P., André S., Gabius H.-J., Jiménez-Barbero J., Leino R. (2012). Studies related to Norway spruce galactoglucomannanes: Chemical synthesis, conformation analysis, NMR spectroscopic characterization, and Molecular recognition of model compounds. Chem. Eur. J..

[56] Pu Y., Jiang N., Ragauskas A.J. (2007). Ionic liquid as a green solvent for lignin. J. Wood Chem. Technol..

[57] Kilpeläinen I., Xie H., King A., Granstrom M., Heikkinen S., Argyropoulos D.S. (2007). Dissolution of wood in ionic liquids. J. Agric. Food Chem..

[58] Xie H., King A., Kilpeläinen I., Granström M., Argyropoulos D.S. (2007). Thorough chemical modification of wood-based lignocellulosic materials in ionic liquids. Biomacromolecules.

[59] Qu C., Kishimoto T., Kishino M., Hamada M., Nakajima N. (2011). Heteronuclear single-quantum coherence nuclear magnetic resonance (HSQC NMR) characterization of acetylated fir (*Abies sachallnensis MAST*). Wood regenerated from ionic liquid. J. Agric. Food Chem..

[60] Giernoth R., Bankmann D., Schlörer N. (2005). High performance NMR in ionic liquids. Green Chem..

[61] Giernoth R. (2009). NMR spectroscopy in ionic liquids. Top. Curr. Chem..

[62] Yelle D.J., Ralph J., Frihart C.R. (2008). Characterization of nonderivatized plant cell walls using high-resolution solution-state NMR spectroscopy. Magn. Res. Chem..

[63] Yoneda Y., Ebner G., Takano T., Nakatsubo F., Potthast A., Rosenau T. (2009). Synthesis of the perdeuterated cellulose solvents 1-ethyl-3-methyl imidazolium acetate (EMIM-OAc-d_14_) and 1-butyl-3-methylimidazolium acetate (BMIM-OAc-d_18_) and of 2-^13^C-butyl-3-methylimidazolium acetate. J. Label. Compd. Radiopharm..

[64] Akim L.G., Argyropoulos D.S., Jouanin L., Leplé J.C., Pilate G., Pollet B., Lapierre C. (2001). Quantitative ^31^P NMR spectroscopy of lignins from transgenic poplars. Holzforschung.

[65] Nimz H.H., Robert D., Faix O., Nemr M. (1981). Carbon-13 NMR spectra of lignins, 8: Structural differences between lignins of hardwoods, softwoods, grasses and compression wood. Holzforschung.

[66] Gagnaire D. (1984). Recent developments in wood chemistry. Actual. Chim..

[67] Gil A.M., Pascoal Neto C. (1999). Solid-state NMR studies of wood and other lignocellulosic materials. Annu. Rep. NMR Spectrosc..

[68] Frazier C.E., Ni J., Schmidt R.G. (1999). Applications of NMR spectroscopy to the analysis of wood/adhesive bondlines. Adv. Lignocellul. Charact..

[69] Atalla R.H., Vander Hart D.L. (1999). The role of solid state 13C NMR spectroscopy in studies of the nature of native celluloses. Solid State Nucl. Magn. Reson..

[70] Maunu S.L. (2002). NMR studies of wood and wood products. Prog. Nucl. Magn. Reson. Spectrosc..

[71] Wikberg H., Maunu S.L. (2004). Characterisation of thermally modified hard- and softwoods by ^13^C CPMAS NMR. Carbohydr. Polym..

[72] Bardet M., Gerbaud G., Giffard M., Doan C., Hediger S., Pape L.L. (2009). 13C high-resolution solid-state NMR for structural elucidation of archaeological woods. Prog. Nucl. Magn. Reson. Spectrosc..

[73] Ralph J., Landucci L.L., Heitner C., Dimmel D.R., Schmidt J.A. (2010). NMR of lignins. Lignin and Lignans; Advances in Chemistry.

[74] Terrett O.M., Lyczakowski J.J., Dinu Iuga L.Y., Franks W.T., Brown S.P., Dupree R., Dupree P. (2019). Molecular architecture of softwood revealed by solid-state NMR. Nat. Commun..

[75] Bardet M., Gagnaire D., Nardin R., Robert D., Vincendon M. (1986). Use of carbon-13 enriched wood for structural NMR investigation of wood and wood components, cellulose and lignin, in solid and in solution. Holzforschung.

[76] Hawkes G.E., Smith C.Z., Utley J.H.P., Vargas R.R., Viertler H. (1993). A comparison of solution and solid state 13 C NMR spectra of lignins and lignin model compounds. Holzforschung.

[77] Pu Y., Hallac B., Ragauskas A.J., Wyman C.E. (2013). Plant biomass characterization: Application of solution and solid-state NMR spectroscopy. Aqueous Pretreatment of Plant Biomass for Biological and Chemical Conversion to Fuels and Chemicals.

[78] Foston M., Samuel R., He J., Ragauskas A.J. (2016). A review of whole cell wall NMR by the direct-dissolution of biomass. Green Chem..

[79] Aoki D., Nomura K., Hashiura M., Imamura Y., Miyata S., Terashima N., Matsushita Y., Nishimura H., Watanabe T., Katahira M. (2019). Evaluation of ring-5 structures of guaiacyl lignin in *Ginkgo biloba* L. using solid- and liquid-state 13C NMR difference spectroscopy. Holzforschung.

[80] Happs R.M., Addison B., Doeppke C., Donohoe B.S., Davis M.F., Harman-Ware A.E. (2021). Comparison of methodologies used to determine aromatic lignin unit ratios in lignocellulosic biomass. Biotechnol. Biofuels.

[81] Schledorn M., Malär A.A., Torosyan A., Penzel S., Klose D., Oss A., Org M.-L., Wang S., Lecoq L., Cadalbert R. (2020). Protein NMR Spectroscopy at 150 kHz Magic—Angle Spinning Continues To Improve Resolution and Mass Sensitivity. ChemBioChem.

[82] Successful Installation of World’s First 1.2 GHz NMR System Enables Novel Functional Structural Biology Research. https://www.bruker.com/en/news-and-events/news/2020/successful-installation-of-worlds-first-12-ghz-NMR-system-enables-novel-functional-structural-biology-research.html.

[83] Wang Z., Yokoyama T., Chang H.-M., Matsumoto Y. (2009). Dissolution of beech and spruce milled woods in LiCl/DMSO. J. Agric. Food Chem..

[84] Drafz M.H.H. (2014). Synthese und Analytik Neuartig Modifizierter Holzoberflächen.

[85] Holz M., Weingärtner H. (1991). Calibration in accurate spin-echo self-diffusion measurements using ^1^H and less-common nuclei. J. Magn. Reson..

[86] Lounila J., Oikarinen K., Ingman P., Jokisaari J. (1996). Effects of thermal convection on NMR and their elimination by sample rotation. J. Magn. Reson. A.

[87] Nakamura I., Nemoto T., Shiraiwa N., Terada M. (2009). Palladium-catalyzed indolization of N-aroylbenzotriazoles with disubstituted alkynes. Org. Lett..

[88] Hill C.A.S., Jones D. (1996). The Dimensional stabilisation of Corsican pine sapwood by reaction with carboxylic acid anhydrides. The effect of chain length. Holzforschung.

[89] Kerssebaum R. (2002). DOSY and Diffusion by NMR. A Tutorial for TopSpin 2.0.

[90] Nilsson M. (2009). The DOSY Toolbox: A new tool for processing PFG NMR diffusion data. J. Magn. Reson..

[91] Cabrita E.J., Berger S. (2001). DOSY studies of hydrogen bond association: Tetramethylsilane as a reference compound for diffusion studies. Magn. Reson. Chem..

[92] Phuong L.X., Takayama M., Shida S., Matsumoto Y., Aoyagi T. (2007). Determination of the accessible hydroxyl groups in heat-treated *Styrax tonkinensis* (Pierre) Craib ex Hartwich wood by hydrogen-deuterium exchange and ^2^H NMR spectroscopy. Holzforschung.

[93] Burger R., Bigler P. (1998). DEPTQ: Distorsionless enhancement by polarization transfer including the detection of quaternary nuclei. J. Magn. Res..

[94] Bigler P., Kümmerle R., Bermel W. (2007). Multiplicity editing including quaternary carbons: Improved performance for the ^13^C-DEPTQ pulse sequence. Magn. Reson. Chem..

[95] Cicero D.O., Barbato G., Bazzo R. (2001). Sensitivity enhancement of a Two-dimensional experiment for the measurement of heteronuclear long-range coupling constants, by a new scheme of coherence selection by gradients. J. Magn. Reson..

[96] Claridge T.D.W., Perez-Victoria I. (2003). Enhanced ^13^C resolution in semi-selective HMBC: A band-selective, constant-time HMBC for complex organic structure elucidation by NMR. Org. Biomol. Chem..

[97] (2020). ACD/C+H NMR Predictors and DB 2020.

[98] Kalinowski H.-O., Berger S., Braun S. (1984). 13C-NMR-Spektroskopie. 200 Tabellen.

[99] Barrelle M. (1993). A new method for the quantitative ^19^F NMR spectroscopic analysis of hydroxyl groups in lignins. Holzforschung.

[100] Barrelle M. (1995). Improvements in the structural investigation of lignins by ^19^F NMR spectroscopy. J. Wood Chem. Technol..

[101] Ahvazi B.C., Argyropoulos D.S. (1996). ^19^F nuclear magnetic resonance spectroscopy for the elucidation of carbonyl groups in lignins.1. Model compounds. J. Agric. Food Chem..

[102] Ahvazi B.C., Crestini C., Argyropoulos D.S. (1999). ^19^F nuclear magnetic resonance spectroscopy for the quantitative detection and classification of carbonyl groups in lignins. J. Agric. Food Chem..

[103] Stejskal E.O., Tanner J.E. (1965). Spin diffusion measurements: Spin echoes in the presence of a time-dependent field gradient. J. Chem. Phys..

