# Supplementary material for: Durable Modification of Wood by Benzoylation—Proof of Covalent Bonding by Solution State NMR and DOSY NMR Quick-Test
Source: Polymers (Basel). 2021 Jun 30;13(13):2164. doi: 10.3390/polym13132164 (PMC8271922; doi:10.3390/polym13132164)
Supplement: Supplementary file 1 [file polymers-13-02164-s001.zip › polymers-1271414-supplementary.pdf]

Supporting Information

Durable Modification of Wood by Benzoylation – Proof of Covalent Bonding by Solution State NMR and DOSY NMR Quick-test

Jan C. Namyslo, Martin H. H. Drafz and Dieter E. Kaufmann*

Table of contents

1	General Information according to NMR measurements	S3
2	Additional NMR spectra of cellulose triacetate (model compound for comparison)	S3
2.1	^{1}H NMR spectrum	S3
2.2	^{13}C-APT (attached proton test)	S4
2.3	gs-^{1}H,^{13}C-HSQC (one-bond correlation)	S5
2.4	gs-^{1}H,^{13}C-HMBC spectrum	S6
3	NMR spectra of 1*H*-benzotriazol-1-yl][3,5-bis(trifluoromethyl) phenyl]methanone	S7
3.1	^{1}H NMR spectrum	S7
3.2	^{13}C NMR spectrum	S7
3.3	^{13}C-DEPT135 spectrum	S8
3.4	^{15}N NMR spectrum	S8
3.5	^{19}F NMR spectrum	S9
3.6	gs-^{1}H,^{1}H-COSY spectrum	S9
3.7	gs-^{1}H,^{13}C-HSQC spectrum	S10
3.8	gs-^{1}H,^{13}C-HMBC spectrum	S10

4	Solution state 1D NMR spectra of 3,5-bis(trifluoromethyl)benzoate derived wood	S11
4.1	^{1}H NMR of chemically modified wood	S11
4.2	^{19}F NMR of chemically modified wood	S11
5	DOSY NMR spectrum of 3,5-bis(trifluoromethyl)benzoic acid and its methyl ester	S12
5.1	^{1}H NMR of chemically modified wood	S12
6	Diffusion rates and DOSY parameters	S12

1 General Information according to NMR spectra

NMR spectra were recorded at room temperature with a Bruker Avance III 600 MHz NMR spectrometer equipped with a broad-band observe (BBO) probe head with z-gradient.

For one-dimensional proton spectra a standard zg30 sequence was used. Corresponding carbon spectra were obtained as DEPTQ (detecting also quaternary carbons) or as attached-proton test (APT). As usual, within these carbon spectra CH_3 and CH groups direct upwards, whereas CH_2 and also quaternary carbons direct downwards related to the baseline.

All 2D NMR spectra of wood-derived analytes were conducted with time-saving non-uniform sampling recording 10% NUS data via a poisson-gap sampling strategy (see main text).

2 NMR spectra of cellulose triacetate (model compound), ca. 5 mg in 600µL $CDCl_3$

2.1 ^{1}H-NMR (600.35 MHz), 16 scans

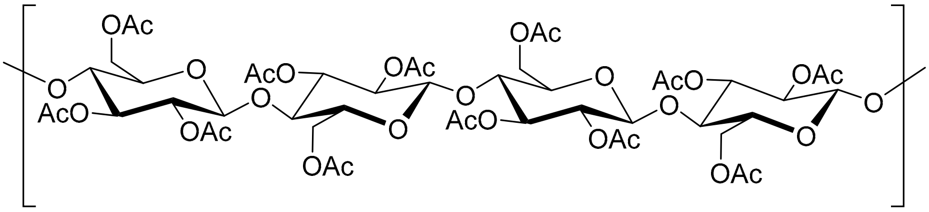

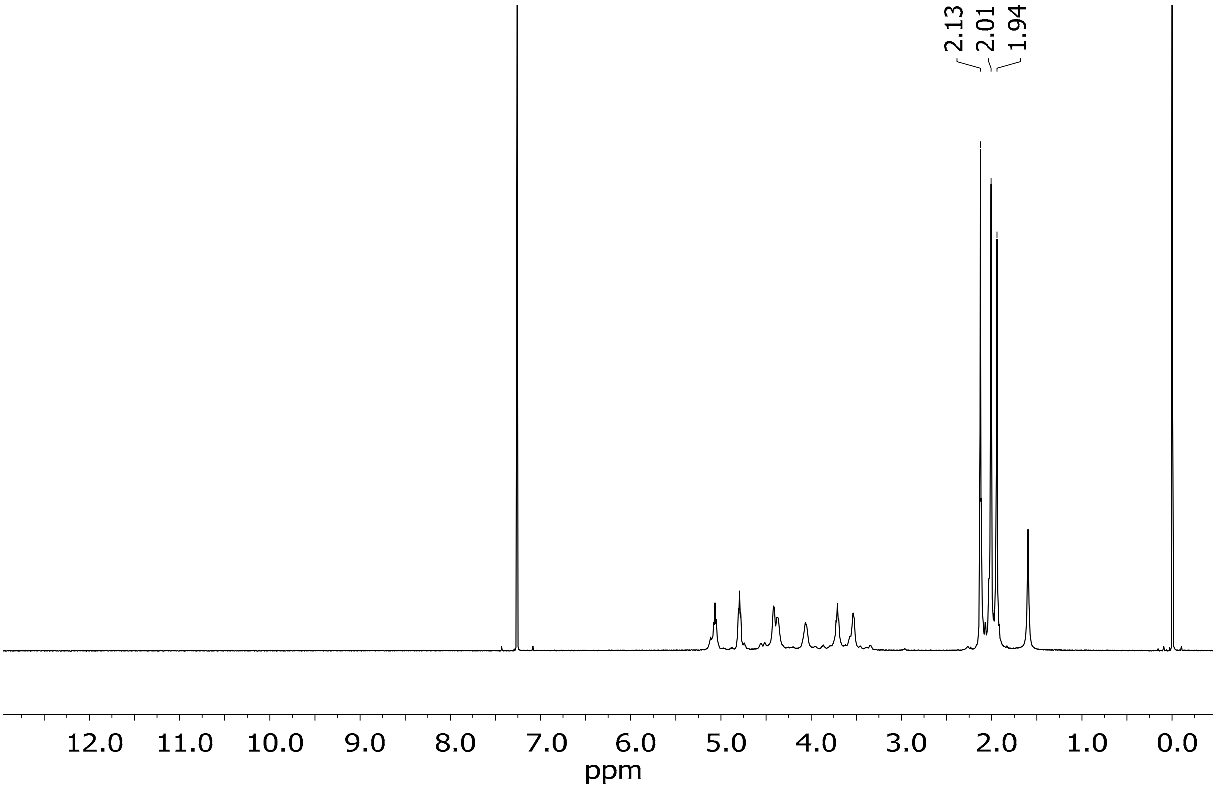

2.2 ^{13}C-APT (150.97 MHz), (attached proton test) of cellulose triacetate, 4k scans

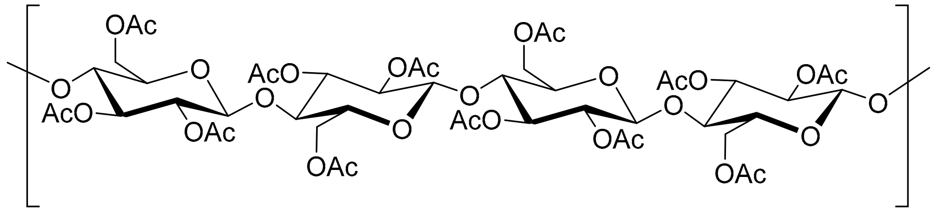

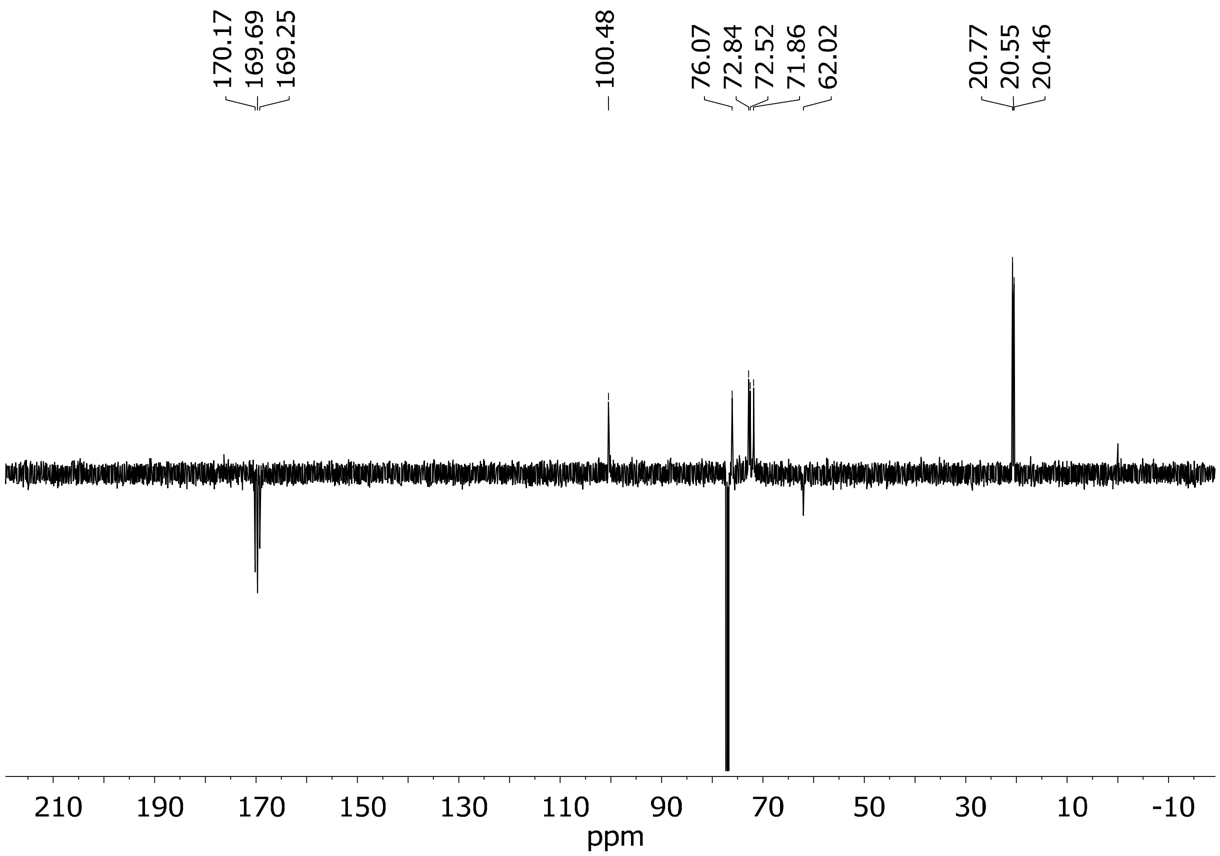

2.3 gs-^{1}H,^{13}C-HSQC (one-bond C-H correlation) of cellulose triacetate, traditional recording (uniform sampling), 512 increments.

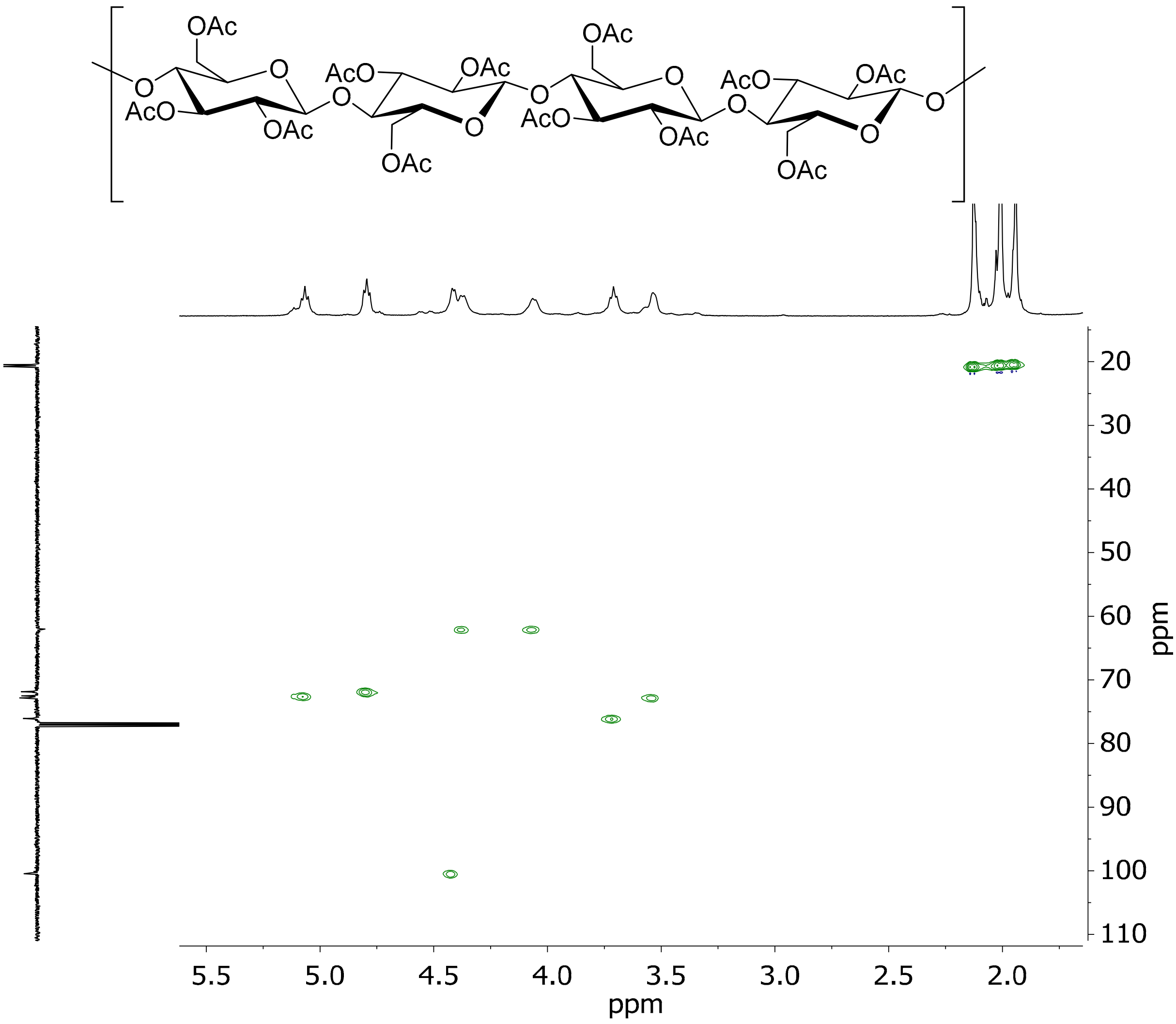

2.4 gs-¹H,¹³C-HMBC (multiple-bond C-H correlation) of cellulose triacetate, traditional recording (uniform sampling), 512 increments.

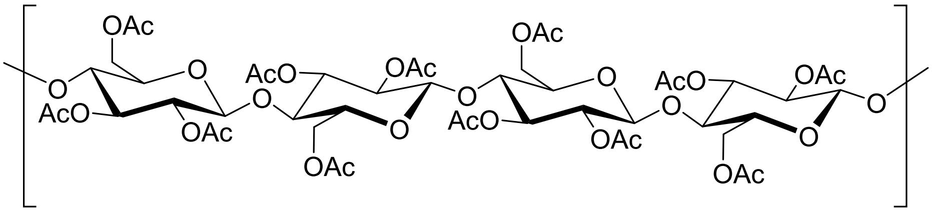

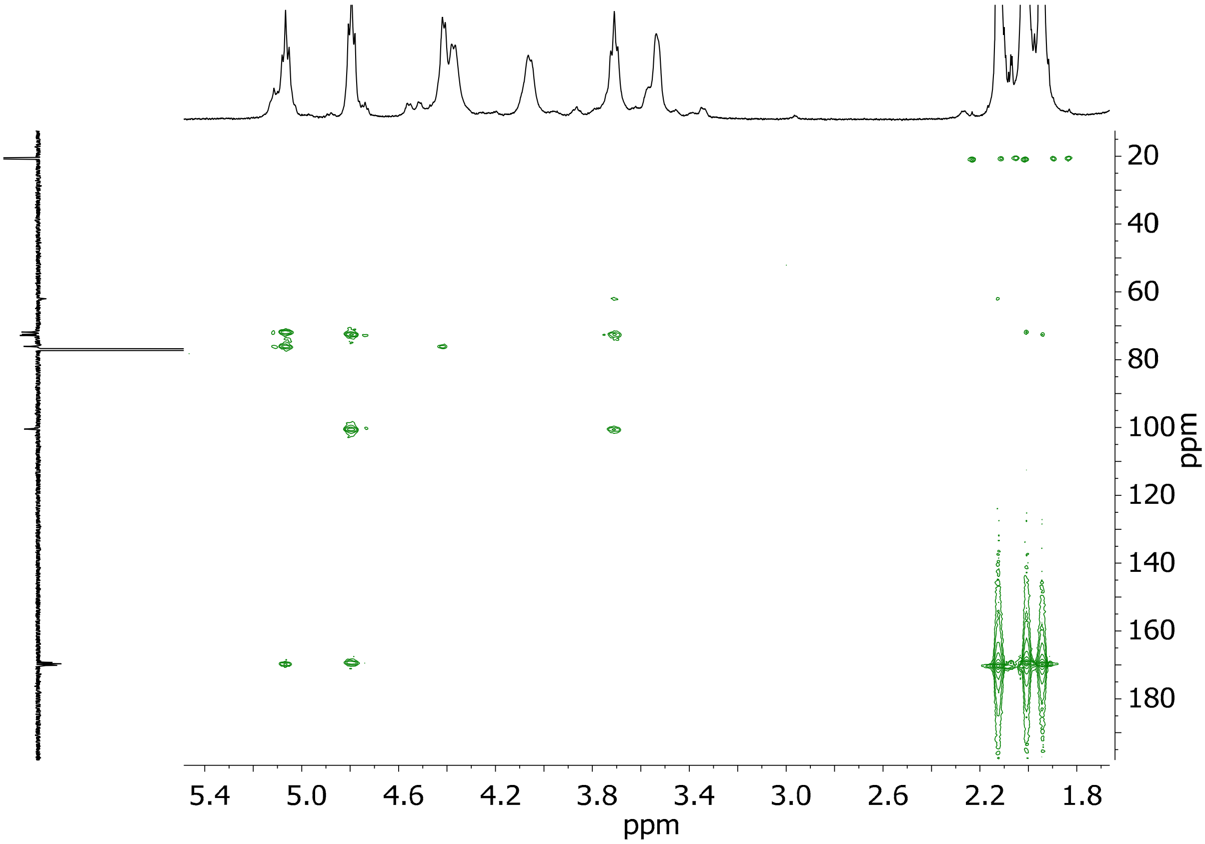

3 NMR spectra of 1*H*-benzotriazol-1-yl[3,5-bis(trifluoromethyl)phenyl]methanone

3.1 ^{1}H-NMR (600.35 MHz),16 scans

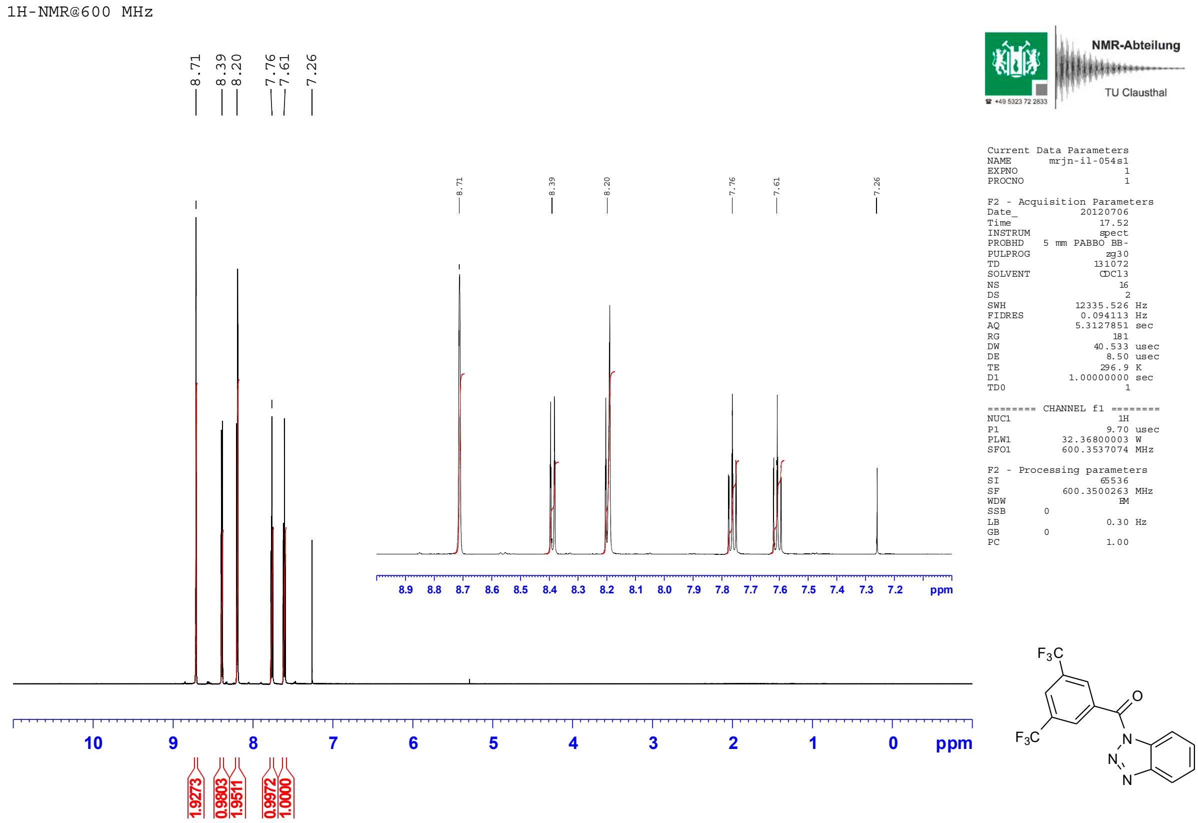

3.2 ^{13}C-NMR (150.97 MHz), zgpg30, 1k scans

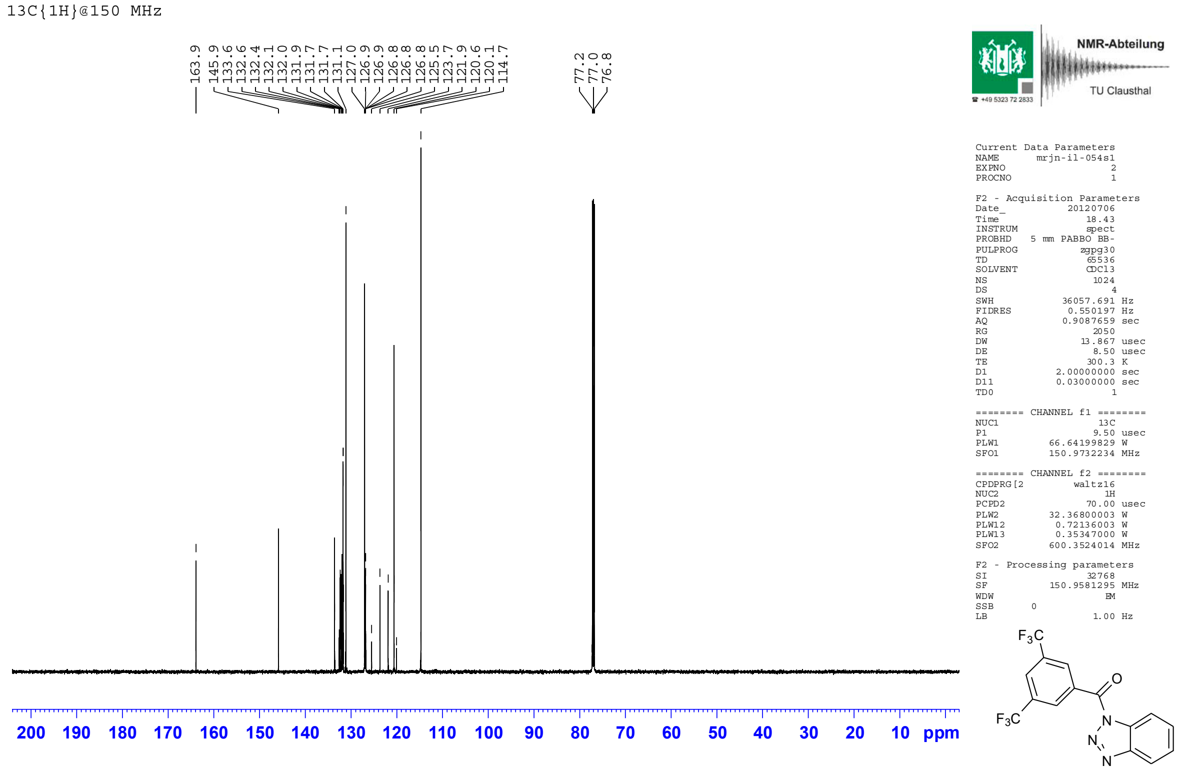

3.3 ^{13}C-DEPT135 NMR (150.97 MHz), dept135, 512 scans

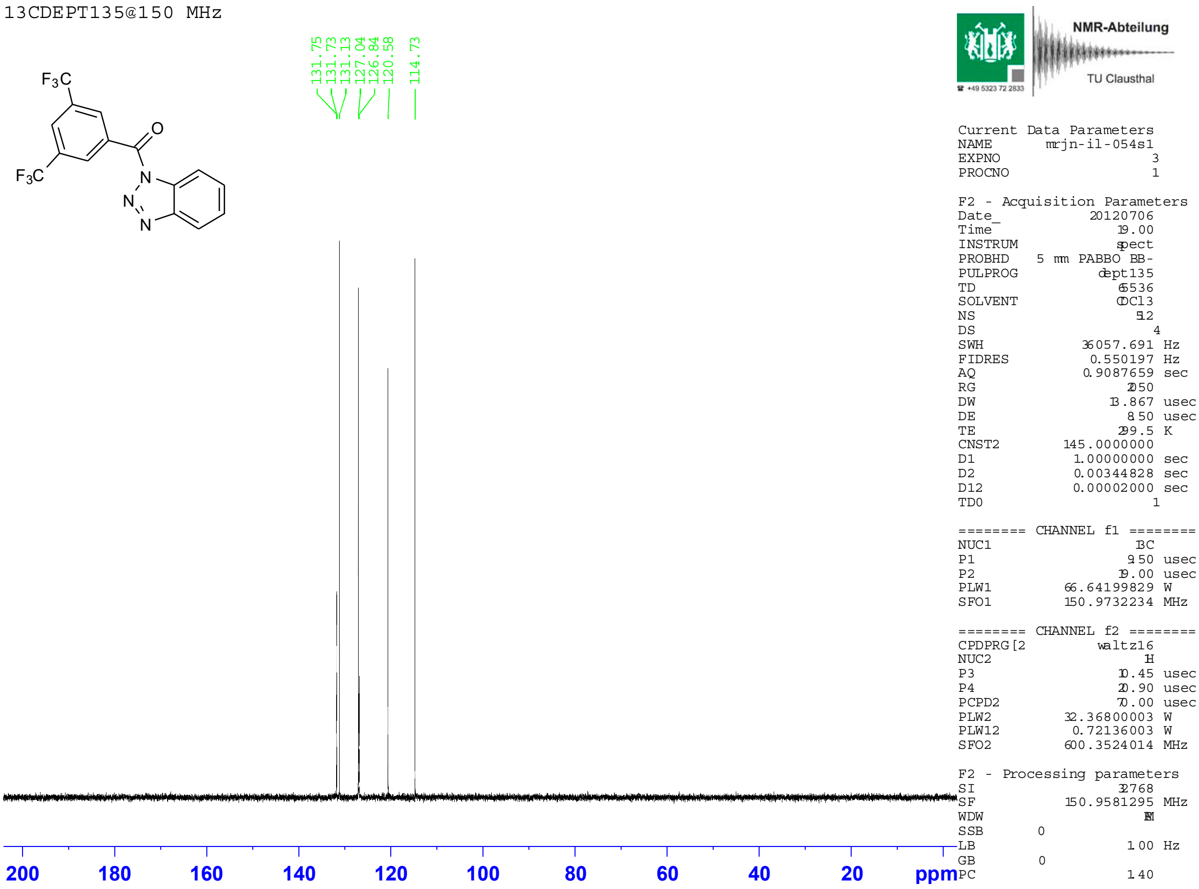

3.4 ^{15}N-NMR (60.85 MHz), zgig30, 16k scans

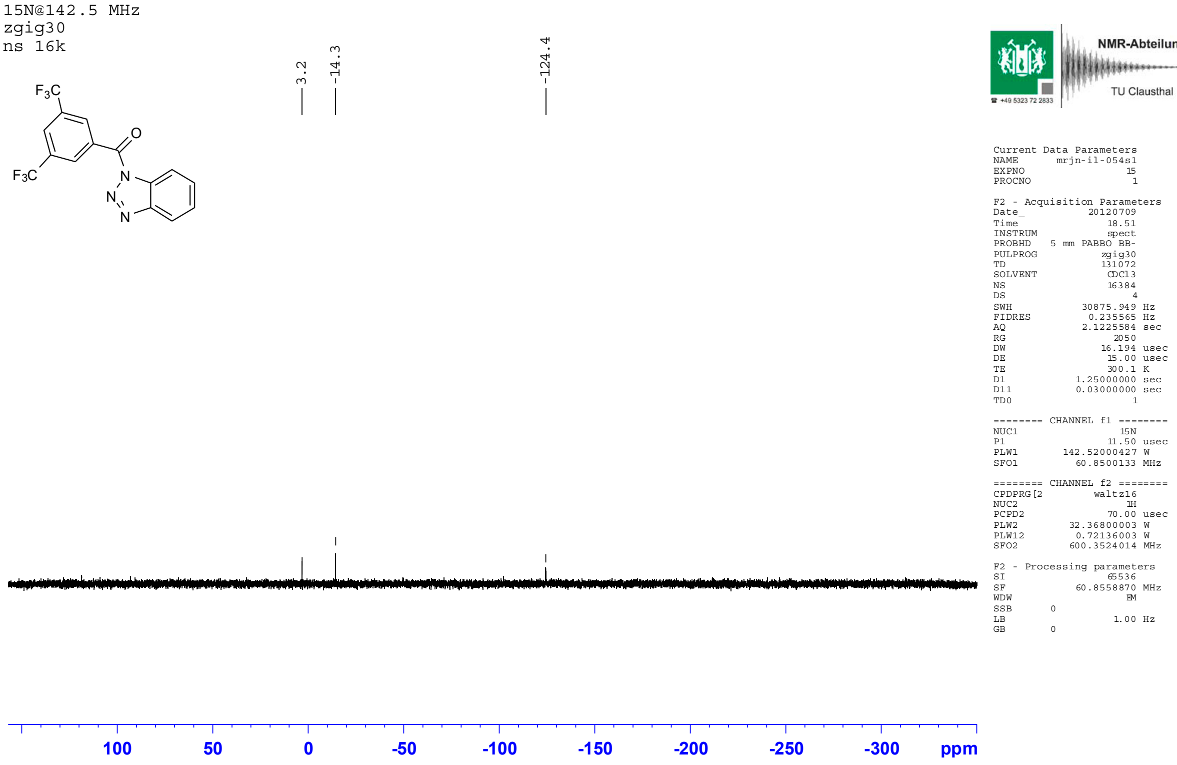

3.5 ^{19}F-NMR (564.83 MHz), zgfhigqn, 16 scans

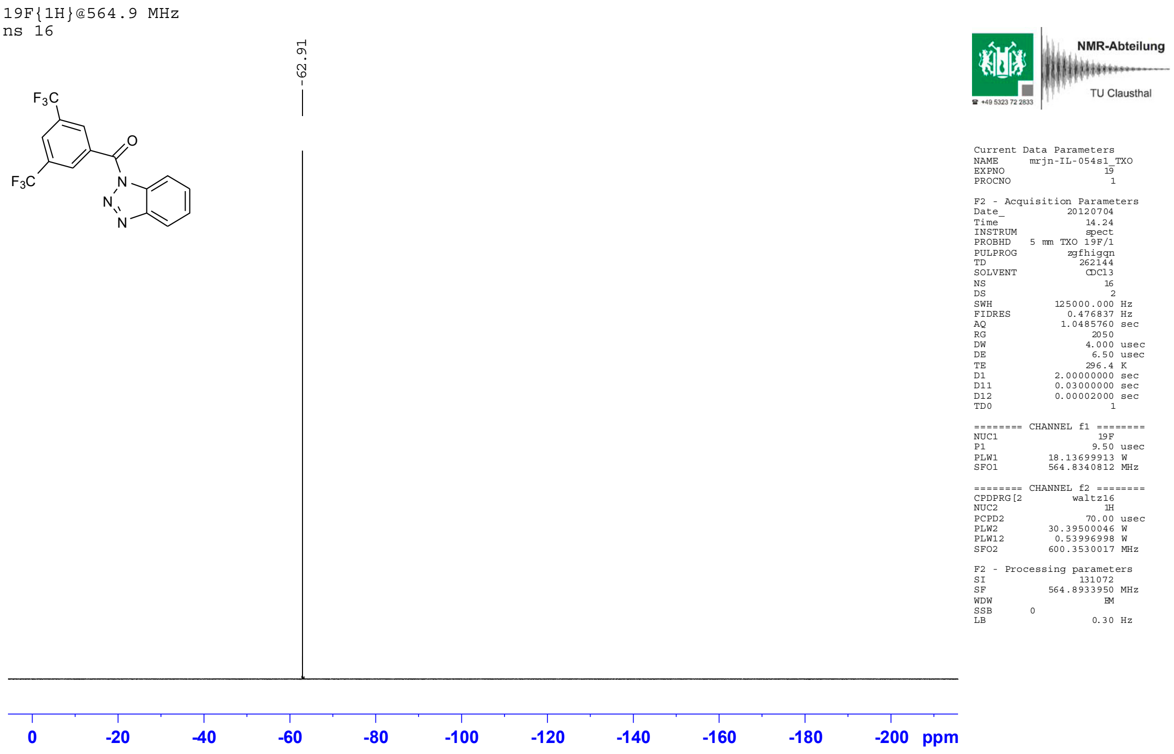

3.6 gs-^{1}H, ^{1}H-COSY (cosygpmfqf)

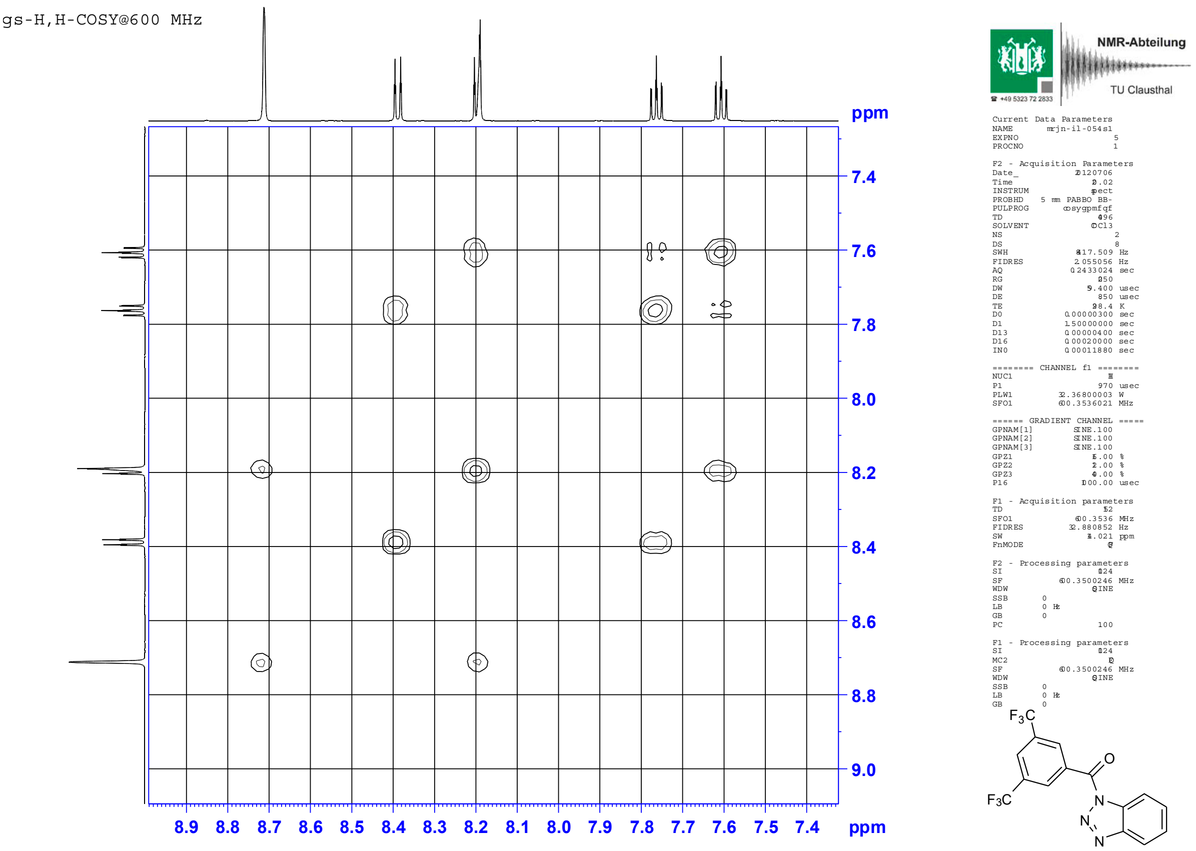

3.7 gs-^{1}H,^{13}C-HSQC (one-bond correlation, pp: hsqcetgp)

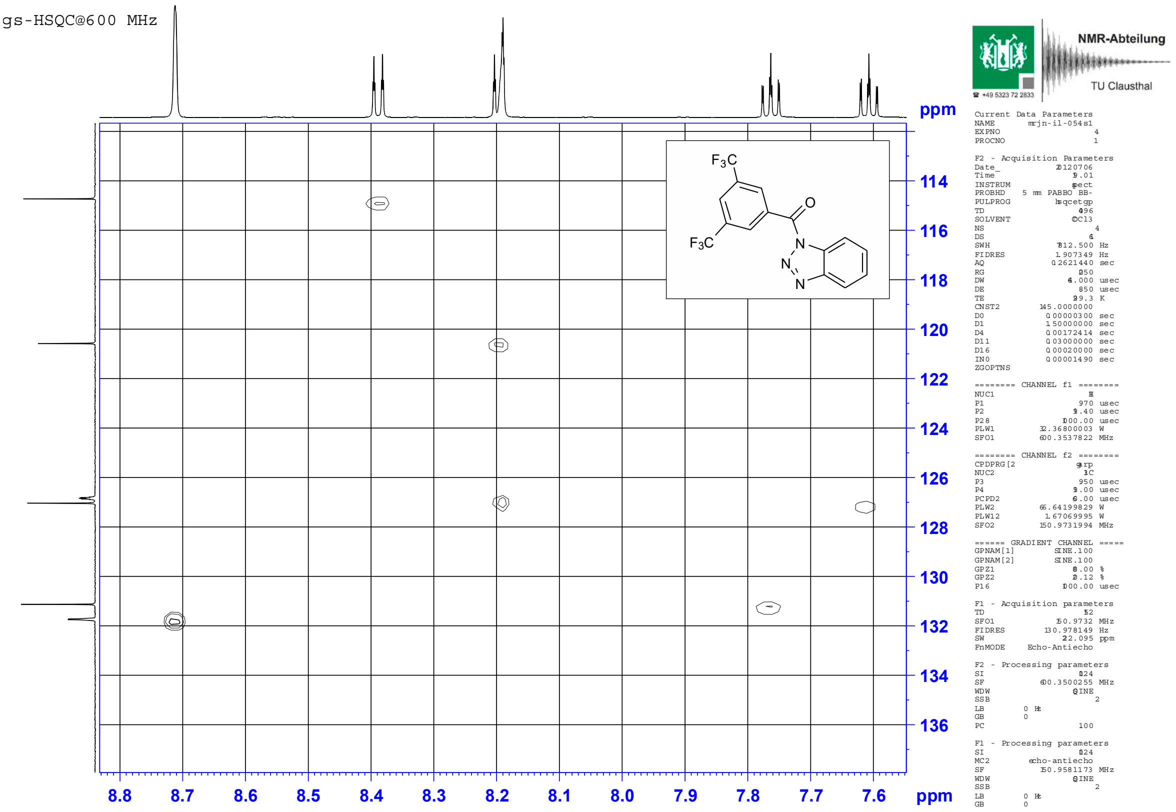

3.8 gs-^{1}H,^{13}C-HMBC (multiple-bond correlation, pp: hmbcgplpndqf)

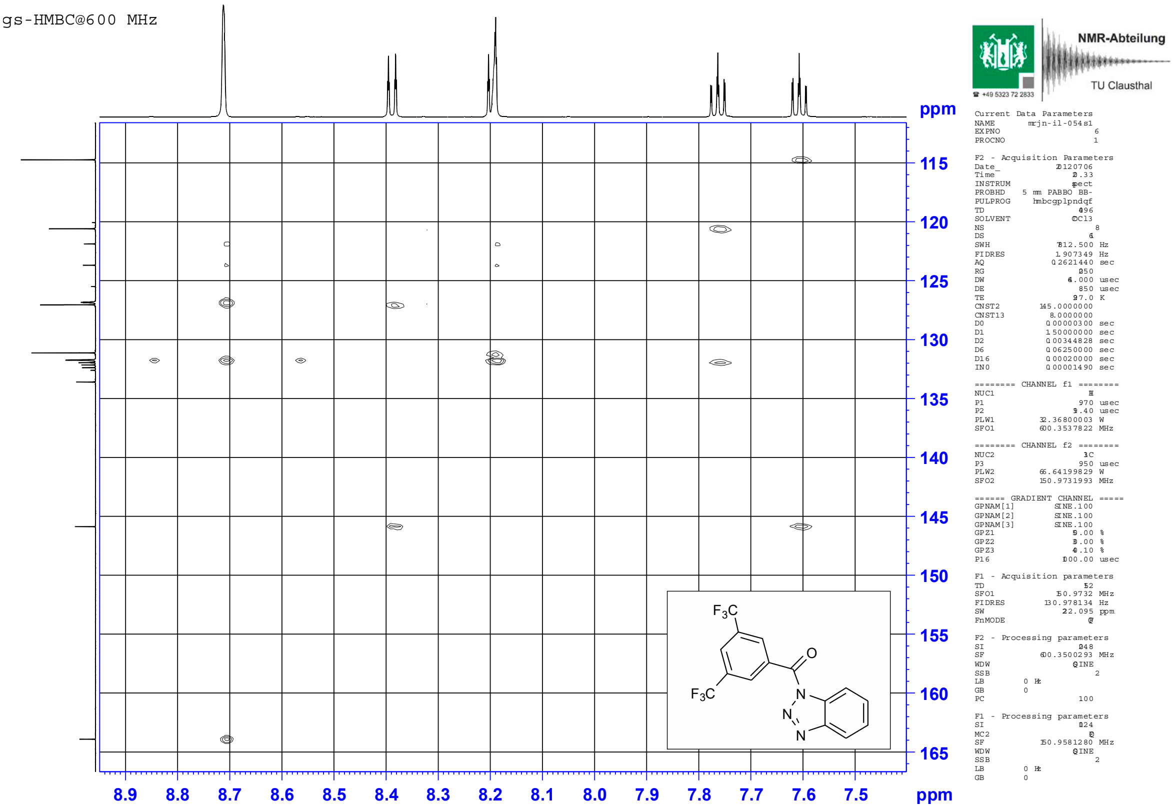

4 Solution state 1D NMR spectra of 3,5-bis(trifluoromethyl)benzoylated wood

4.1 ^{1}H-NMR (600.35 MHz) of 3,5-bis(trifluoromethyl)benzoylated wood, 64 scans

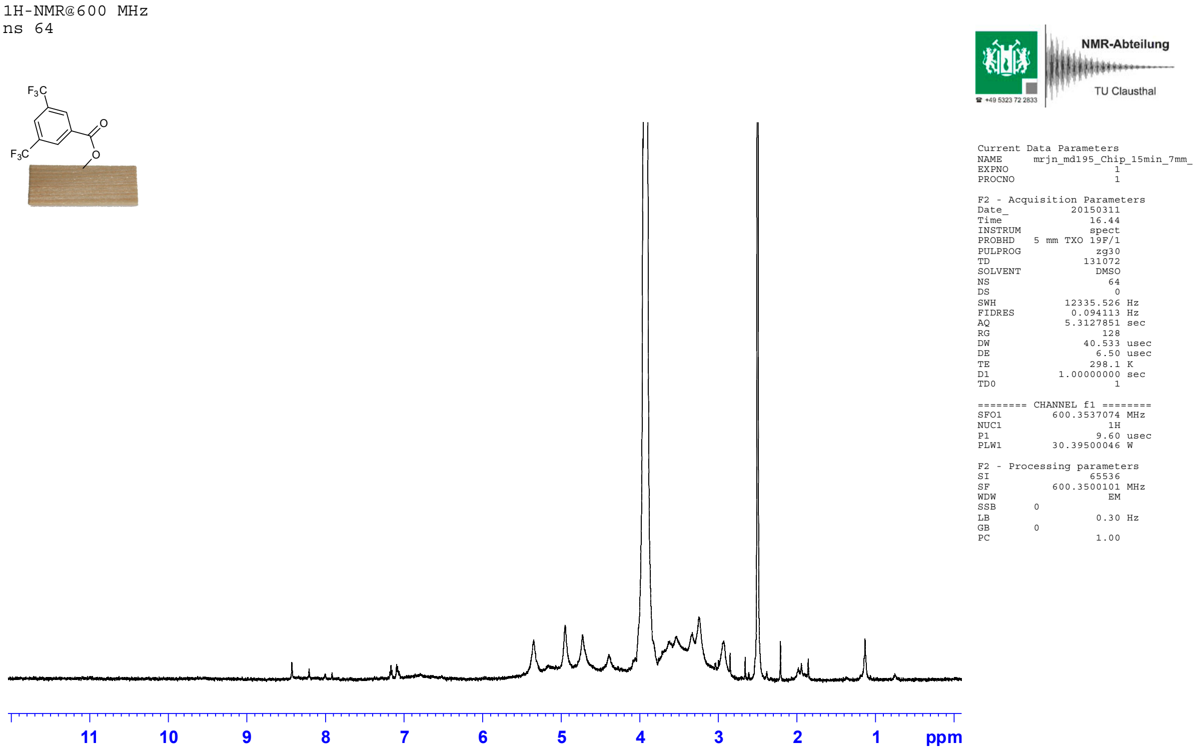

4.2 ^{19}F-NMR (564.83 MHz) of 3,5-bis(trifluoromethyl)benzoylated wood, 64 scans

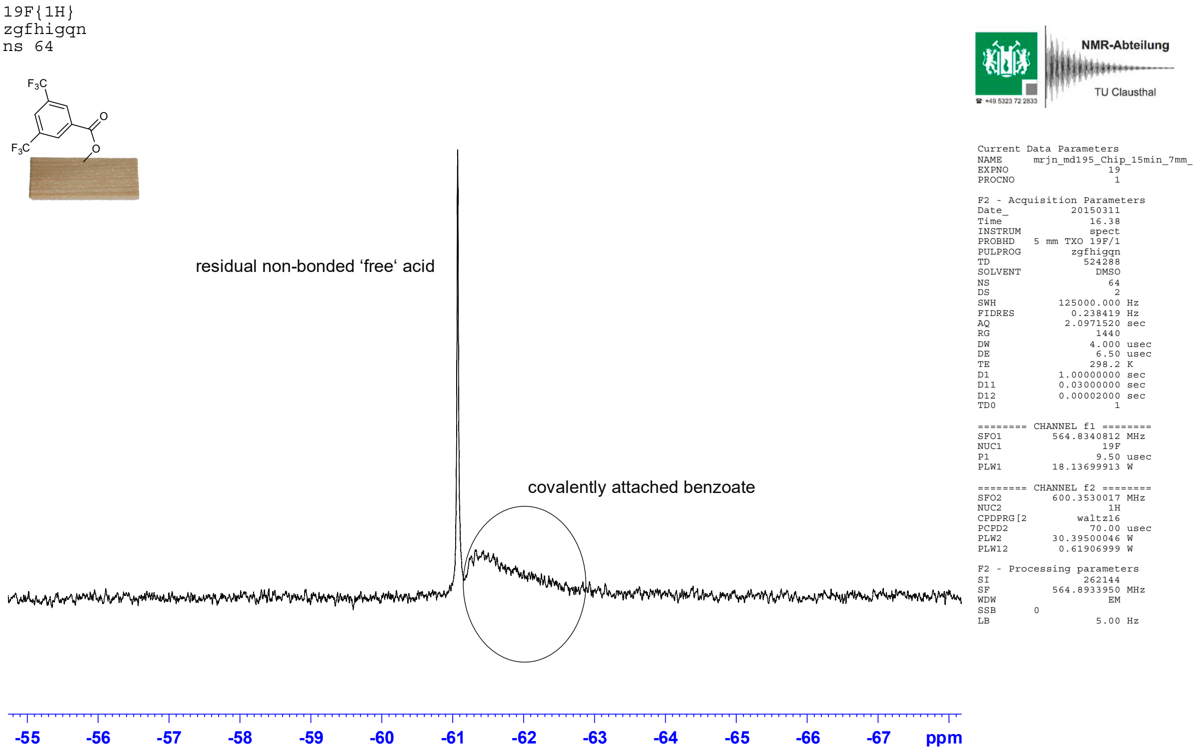

5 DOSY NMR spectrum of 3,5-bis(trifluoromethyl)benzoic acid and its **methyl ester** in DMSO-d_6/LiCl

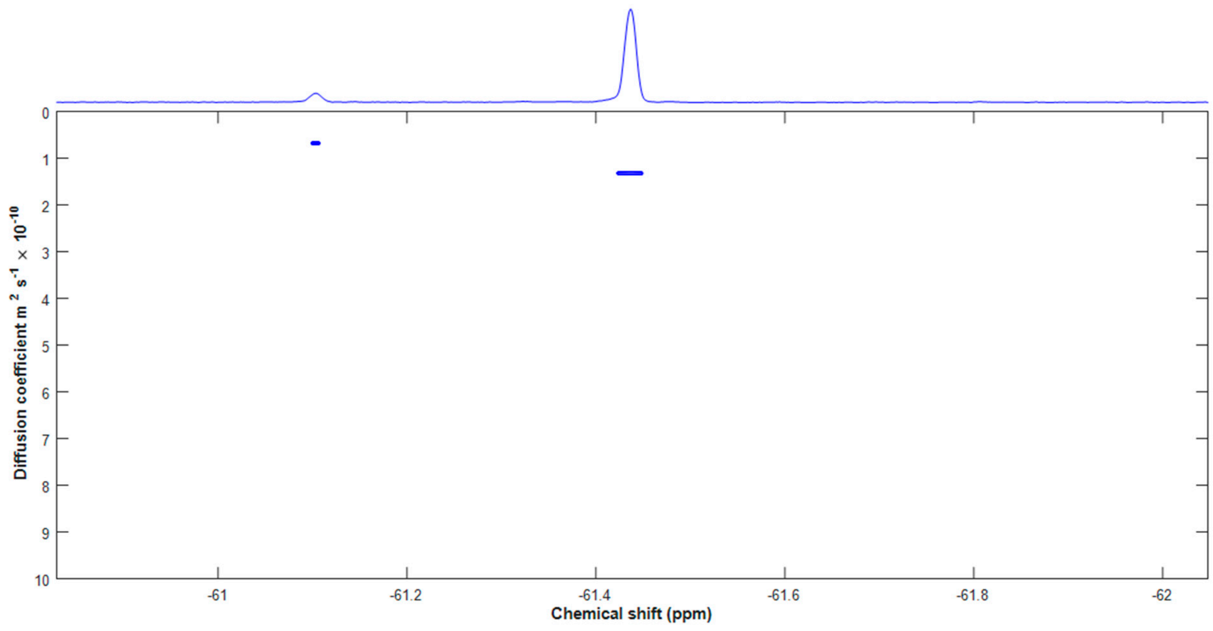

6 Diffusion rates and DOSY parameters (in addition to Table 1 from main text)

entry	compound	solvent	D value [10^{-10} $m^2 \cdot s^{-1}$]
1	3,5-$(CF_3)_2C_6H_3$-COO-**wood**[a]	LiCl/DMSO-d_6	
	^{19}F - DOSY		0.120±0.012[b,d]
	Diffusion time (intergradient-delay, Bruker parameter d20): 750 ms<br>Gradient length (Bruker parameter p30): 4000 µs		
2	^{1}H - DOSY		0.121±0.005[c]
	Diffusion time (intergradient-delay, Bruker parameter d20): 500 ms<br>Gradient length (Bruker parameter p30): 3500 µs		
3	cellulose triacetate	LiCl/DMSO-d_6	0.028±0.002[c]
	^{1}H - DOSY		
	Diffusion time (intergradient-delay, Bruker parameter d20): 1000 ms<br>Gradient length (Bruker parameter p30): 4250 µs<br>Optimized pulse repetition delay (d1): 14.4s		

4 cellulose triacetate DMSO-d_6 0.081±0.002[c]

^1H - DOSY

Diffusion time (intergradient-delay, Bruker parameter d20): 500 ms
Gradient length (Bruker parameter p30): 3600 µs

5 TMS LiCl/DMSO-d_6 2.33±0.04[c]

^1H - DOSY

Diffusion time (intergradient-delay, Bruker parameter d20): 100 ms
Gradient length (Bruker parameter p30): 1750 µs

6 TMS DMSO-d_6 5.85±0.02[c]

^1H - DOSY

Diffusion time (intergradient-delay, Bruker parameter d20): 100 ms
Gradient length (Bruker parameter p30): 1100 µs

7 3,5-$(CF_3)_2C_6H_3$-COOH LiCl/DMSO-d_6 0.71±0.00(6)[b]

^{19}F - DOSY

Diffusion time (intergradient-delay, Bruker parameter d20): 180 ms
Gradient length (Bruker parameter p30): 2200 µs

8 3,5-$(CF_3)_2C_6H_3$-COOH DMSO-d_6 3.59±0.04[b]

^{19}F - DOSY

Diffusion time (intergradient-delay, Bruker parameter d20): 120 ms
Gradient length (Bruker parameter p30): 1200 µs

9 3,5-$(CF_3)_2C_6H_3$-COOMe LiCl/DMSO-d_6 1.22±0.06 [b]

^{19}F - DOSY

Diffusion time (intergradient-delay, Bruker parameter d20): 180 ms
Gradient length (Bruker parameter p30): 2200 µs

10	3,5-$(CF_3)_2C_6H_3$-COOMe	DMSO-d_6	4.45±0.10 [b]

^{19}F - DOSY

Diffusion time (intergradient-delay, Bruker parameter d20): 120 ms
Gradient length (Bruker parameter p30): 1200 µs

[a] about 1.7 mg of chemically modified wood (as a flour resulting from mild ball-milling) in 170 µL NMR solvent

[b] ^{19}F-DOSY, c: ^{1}H-DOSY, d: additionally detected free acid gave a D value of about 0.79 10^{-10} $m^2 \cdot s^{-1}$
